# Not just form, not just meaning: Words with consistent form-meaning mappings are learned earlier

**DOI:** 10.1177/17470218211053472

**Published:** 2021-10-21

**Authors:** Giovanni Cassani, Niklas Limacher

**Affiliations:** Department of Cognitive Science and Artificial Intelligence, Tilburg School of Humanities and Digital Sciences, Tilburg University, Tilburg, The Netherlands

**Keywords:** Age of acquisition, form-meaning systematicity, orthography-semantics-consistency, phonology-semantics-consistency, computational psycholinguistics

## Abstract

By leveraging Phonology-to-Semantics Consistency (PSC), which quantifies form-meaning systematicity as the semantic similarity between a target word and its phonological nearest neighbours, we document a unique effect of systematicity on Age of Acquisition (AoA). This effect is also found after controlling for the effect of neighbourhood density measured for word forms and lexical semantics and several other standard predictors of AoA. Moreover, we show that the effect of systematicity is not reducible to iconicity. Finally, we extensively probe the reliability of this finding by testing different statistical models, analysing systematicity in phonology and orthography and implementing random baselines, reporting a robust, unique negative effect of systematicity on AoA, such that more systematic words tend to be learned earlier. We discuss the findings in the light of studies on non-arbitrary form-meaning mappings and their role in language learning, focusing on the analogical process at the interface of form and meaning upon which PSC is based and how it could help children infer the semantics of novel words when context is scarce or uninformative, ultimately speeding up word learning.

## Introduction

The factors that influence how children learn their first language from exposure have been widely studied (Bates & MacWhinney, 1987; Pinker, 1984). The spate of studies which addressed this question highlighted several factors which predict Age of Acquisition (AoA), including word frequency (Braginsky et al., 2019; Ghyselinck et al., 2004), phonological neighbourhood density (PND; Jones & Brandt, 2019), semantic neighbourhood density (SND; Fourtassi et al., 2020), contextual diversity (Hills et al., 2010), perceptual features (Peters & Borovsky, 2019), valence, concreteness, and word length (Braginsky et al., 2019), salience in time, space, and, linguistic context (Roy et al., 2015), iconicity (Laing, 2019; Perry et al., 2015; Sidhu et al., 2021), sound symbolism (Imai & Kita, 2014; Kantartzis et al., 2011), and systematicity in form-meaning mappings (Monaghan et al., 2012, 2014). Building on recent studies that quantified the degree of form-meaning systematicity for individual lexical items (Hendrix & Sun, 2020; Marelli & Amenta, 2018), in this work, we assess the effect of systematicity on AoA, after controlling for known predictors of AoA itself. We are the first to assess the effect of form-meaning systematicity on AoA while controlling for measures of neighbourhood density in form and meaning as well as their interaction. Moreover, we rely on measures of form-meaning systematicity which have been already shown to reliably predict reaction times (RTs) in lexical decision studies as well as morphological priming studies (Amenta et al., 2017, 2020; Hendrix & Sun, 2020; Marelli & Amenta, 2018; Marelli et al., 2015) and reading times in a naturalistic setting (Amenta et al., submitted). Finally, our work is the first to sketch a mechanistic account of how systematicity could facilitate word learning, grounded in analogical processes at the interface between form and meaning (Marelli & Amenta, 2018).

The study of different aspects of the relation between word form and meaning has seen a huge increase over the last decades (see Dingemanse et al., 2015; Nielsen & Dingemanse, 2020; Sidhu & Pexman, 2018a, for reviews about this line of work). Contrary to the long-held position that natural languages need to be arbitrary to be effective communication tools (Hockett, 1960), numerous studies have shown the widespread presence of systematic relations between word form and meaning at the sub-morphemic level, painting a more similar picture to the original position held by de Saussure (1916) that, while being a cardinal organising principle of natural languages, arbitrariness is not absolute and form-meaning systematicity is present beyond morphology. Onomatopoeias, for example, are words that have a direct form-meaning relation because they imitate natural sounds (see Laing, 2019, and references therein for a more detailed discussion of onomatopoeias). Other well-known examples come from studies on sound symbolism (Hinton et al., 1994): in *sound-size symbolism*, for instance, the pseudowords *mil* and *mal* are typically associated with small and large objects, respectively (Sapir, 1929). In *sound-shape symbolism*, on the contrary, people reliably associate the pseudowords *maluma* and *bouba* with rounded, blobbish shapes while *takete* or *kiki* tend to be paired with spiky shapes (Köhler, 1947; Ramachandran & Hubbard, 2001), an effect also observed in pre-literate children (Maurer et al., 2006). Other studies have shown that words contain sub-morphemic cues to their lexical category (Farmer et al., 2006; Fitneva et al., 2009; Kelly, 1992; Sharpe & Marantz, 2017; Wright Cassidy & Kelly, 2001) and that these correspondences may help in bootstrapping lexical category acquisition (Christophe et al., 1997; Fitneva et al., 2009; Monaghan et al., 2007; Morgan & Demuth, 2014), their semantics (Reilly et al., 2012) or both (Cassani et al., 2020). One of the most studied examples is that of phonaestemes, sound-sequences that reliably, but not deterministically, cue a certain semantic dimension: for example, in English, gl- is often found in words related to light such as gl*are*, gl*itter*, and gl*ow* (Bergen, 2004), although this relation is only statistical (consider gl*ue* or gl*ucose*, where the same sound sequence is found in spite of no relation to light). Similarly, Louwerse and Qu (2017) showed that an English word’s valence can be predicted based on a single phonological feature (nasals in word onset).

A useful distinction to be made when discussing non-arbitrariness in natural languages is that between iconicity, which studies resemblances between form and meaning (Blasi et al., 2016; Perry et al., 2015), and *systematicity*, which is concerned with the study of statistical regularities within a language which can be used to predict certain semantic and functional characteristics of words (Cassani et al., 2020; Monaghan et al., 2014). In this work, we focus our attention on the latter aspect and ensure that any relation between systematicity and AoA that we may uncover is not confounded by iconicity.

The study of the relation between systematicity and AoA is also not new. Gasser (2004) pointed out that while arbitrariness is useful when communicating, as it makes potentially confusable lexical items more discriminable by orthogonalising form and context, systematicity could help in learning the language. In the extreme, learning a perfectly arbitrary language is harder because no analogies can be formed and every item has to be learned individually. On the contrary, a completely systematic language would be prone to confusion, since similar words would denote similar concepts, which are likelier to occur in similar contexts, making them harder to discern (Eco, 1995). In line with this argument, it has been shown that when few words are known, systematicity helps map forms to referents, whereas with larger vocabularies, systematicity only supports a more effective learning of category structures (Brand et al., 2018; Monaghan et al., 2011, 2012). Moreover, it has been shown that sound-meaning mappings in the English vocabulary are more systematic than would be expected by chance (Monaghan et al., 2014), suggesting that systematicity is not simply due to pockets of sound symbolism and phonaesthemes, but rather is a more general property found across the entire language. The aforementioned studies, however, did not consider the potentially confounding effects of neighbourhood density at the phonological and semantic level on acquisition patterns, with both PND and SND having been recently shown to influence language acquisition (Fourtassi et al., 2020; Jones & Brandt, 2019). It could thus be the case that the effect of systematicity on acquisition is reduced to that of neighbourhood density in form and semantics, such that words are learned earlier simply because they happen to be deeply entrenched in the language network at multiple levels (Hills et al., 2009) rather than because their form cues their meaning in a reliable way.

Closely related to the goal of assessing whether form-meaning systematicity explains unique variance in AoA above and beyond neighbourhood density in form and meaning, we also explore whether there is an interaction between systematicity and SND. Following the hypothesis by Gasser (2004) that systematicity can harm communication efficacy due to the increased confusability of similar concepts that would also have similar forms, it would be expected that words found in sparser semantic neighbourhoods can afford less arbitrary form-meaning relations because they have a lower risk of being confused for semantically similar words. In line with this hypothesis, Sidhu and Pexman (2018b) reported that words with sparser semantic neighbourhoods were more iconic, even after partialling out the relation between AoA and iconicity. Thus, we first check whether we replicate this finding with systematicity rather than iconicity. Then, we ask ourselves whether SND and form-meaning systematicity interact: Is it the case that the effect of systematicity on AoA is modulated by SND?

Next to contributing to the study of the relation between form-meaning systematicity and AoA, we also set out to extend and validate the use of Phonology-to-Semantics-Consistency (PSC; Amenta et al., 2017), as well as its orthographic counterpart, Orthography-to-Semantics Consistency (OSC; Marelli et al., 2015), to study AoA dynamics. OSC and PSC (to which we will collectively refer as Form-to-Semantics Consistency [FSC]) are two measures of form-meaning systematicity which have been recently shown to predict RTs in lexical decision and morphological priming tasks (Hendrix & Sun, 2020; Marelli & Amenta, 2018) as well as reading times in naturalistic reading (Amenta et al., submitted). Both quantify form-meaning systematicity as the semantic similarity between a target word and its orthographic/phonological nearest neighbours, thus combining the similarity structure of a word in both form and semantic space. Since these measures have been shown to reliably predict lexical processing in a variety of tasks, we aim to probe whether they can also illuminate our understanding of acquisition dynamics.

OSC was first introduced to explain a consistent, yet overlooked, effect in morphological masked priming studies: Participants are faster at recognising stems from transparent sets (e.g., *farm*) in comparison to stems from opaque sets (e.g., *fruit*), regardless of the preceding primes. To explain this effect, Marelli and colleagues (2015) found that orthographic strings that are consistently mapped to similar meanings (i.e., words including the stem *gold*, such as *golden*, *goldfield*, *goldmine*, tend to be connected to the concept *gold*, being semantically more coherent) are more effective in reducing the uncertainty in the activated semantic system. In contrast, strings with less consistent form-to-meaning mappings (i.e., the string *rice* appears in *price*, *tricep*, *licorice*, all of which have looser semantic relations with the concept *rice*) are more challenging to process. OSC was thus developed to quantify the degree of semantic similarity between a target string and its orthographic neighbours, defined as the words which embed the target string. In a subsequent paper, Amenta and colleagues (2017) investigated whether phonology may also activate the semantic system and developed PSC, which uses the same definition as OSC, but is based on the word’s phonological rather than orthographic representations. Since some sub-lexical English strings are pronounced differently (e.g., *ough* in *cough*, *thought*, *plough*), PSC is clearly differentiated from OSC. Both measures were found to be relevant predictors of RTs in lexical decision experiments, such that words with high OSC and high PSC were easier to recognise (Amenta et al., 2017).

FSC measures considering neighbours which embed the target word, however, are primarily geared towards assessing form-meaning systematicity at the level of morphology, which is typically explicitly excluded in studies on form-meaning systematicity. In a more recent study, Hendrix and Sun (2020) investigated the effects of several variables in a lexical decision task, targeting both words and non-words. Having to establish a measure of form-meaning consistency for non-words, which are unlikely to be embedded in other words in the lexicon, they defined form-based neighbours as the words with the lowest Levenshtein edit-distance^1^ from the target word (Levenshtein, 1966). This measure was shown to predict RTs in a lexical decision task for both words and non-words, above and beyond other covariates. FSC measures based on Levenshtein distance sidestep the morphological relations between stems and derived words. This formulation of FSC may thus be more informative to capture non-arbitrary patterns that bear relevance in acquisition and are less confounded by morphological productivity. The comparison of these two implementations of FSC will therefore be interesting to check whether a relation between systematicity and AoA is primarily driven by morphological regularities or by patterns with less obvious correlates in morphology. To control for systematicity based on morphology, we also control for morphological complexity, since many of the first words children learn are monomorphemic (Clark, 2017).

Next to predicting RTs in lexical decision tasks, OSC was also recently found to reliably predict reading times (Amenta et al., submitted) and to interact with surprisal in speeding up reading when the context was not sufficiently constraining in guiding prediction. Therefore, in this work, we take a measure to compute word-level form-meaning systematicity which has been shown to be a useful tool to investigate psycholinguistic phenomena such as reading patterns and lexical decision, and apply it to the analysis of acquisition patterns. If the same measure would prove to be a reliable predictor of AoA, we would thus have a single, effective tool to further analyse the role of form-meaning systematicity not just in lexical processing but also in word learning. Moreover, FSC can be seen as a mechanistic account of how to derive semantic representations from word form alone through an analogical process. Upon the first encounter with a novel word, possibly in an uninformative situational or linguistic context, learners could still gauge the semantics of this new word by leveraging the semantics of similar words they already know. If FSC reliably predicts AoA, we would have preliminary evidence that words for which the process of estimating lexical semantics from word form is more reliable and informative (reflected in higher systematicity) are learned earlier. This could mean that children indeed make educated guesses about lexical semantics by exploiting word forms, relying on systematic correspondences between form and meaning, and that learning benefits from situations where this guesstimation is more reliable, resulting in more systematic words being learned earlier.

From a methodological perspective, the approach underlying FSC is not dissimilar from the one introduced by Otis and Sagi (2008), but broadens its scope and explores different ways of assessing form similarity. Whereas Otis and Sagi (2008) used a similar approach to assess the strength of phonaesthemes, considering as orthographic neighbours all words which contained a letter sequence which was a candidate phonaestheme, FSC measures form-meaning systematicity considering less constrained similarities in form. Different approaches to the study of phonaesthemes using measures of form similarity and distributed semantic representations similar to Otis and Sagi (2008) and to FSC were also adopted by Liu and colleagues (2018) and Abramova and colleagues (2013). All measures were shown to capture known phonaesthemes while not falling prey to similarities in form which are not recognised as phonaesthemic. Moreover, these methods could also find new candidate phonaesthemes, suggesting that the general approach can inform our understanding of non-arbitrariness in language. These results further strengthen the viability of a measure grounded in analogical processes at the interface of form and meaning, which our study aims to extend from the study of systematicity per se to the analysis of its influence on language acquisition.

FSC measures are however not the only way in which form-meaning systematicity has been assessed in the literature. The most straightforward way to assess systematicity is to measure whether items with similar forms also tend to have similar semantic representations, thus measuring the correlation between pairwise similarities across different representations for the same words (Dautriche et al., 2017; Monaghan et al., 2014; Shillcock et al., 2001; Tamariz, 2008). All studies controlled for the effect of morphology by restricting attention to monomorphemic words and reported small yet significant correlations across several languages. In particular, Monaghan and colleagues (2014) quantified the degree of systematicity of each lexical item by measuring how the correlation between pairwise similarities in form and meaning changed when excluding each target word. The difference between the correlation computed including and excluding a given target word was taken to reflect the word’s degree of systematicity. This study shows that more systematic words tend to be overrepresented among early acquired words, pointing to a relation between systematicity and acquisition.

More recently, neural network methods have also been used to quantify systematicity (Pimentel et al., 2019). First, word forms were encoded using a deep learning model such that each word was represented as a probabilistic distribution over discrete units, i.e., letters. Then, a different model was trained to reconstruct the word form conditioned on the semantic representation of the word. The entropy between the two distributions, the true, unconditioned one and the one reconstructed on the basis of semantic information, was taken to reflect the degree of form-meaning systematicity of each word. A small yet reliable systematicity was found for a large number of typologically different languages.

These methods, however, are rather computationally expensive as compared to FSC measures, and crucially, only work for words for which a semantic representation estimated from language data is available, thus speaking only about a mature system and not being able to say much about learning patterns. Recent work has however started to show that systematicity is fruitfully studied using computational methods also for pseudowords (Baayen et al., 2019; Cassani et al., 2020; Chuang et al., 2021; Hendrix & Sun, 2020). In particular, OSC has been used by Hendrix and Sun (2020) to assess the semantic coherence of the form-based nearest neighbours of words and pseudowords alike. This is particularly interesting since, at some point in development, any word is a pseudoword to language learners, who need to be able to map the new word to a semantic representation: FSC could help illuminate this task, by sketching a process where children leverage word form and analogies across form and meaning to estimate lexical semantics even without an informative situational and linguistic context.

To sum up, this work leverages and extends two different yet connected lines of research. On one hand, we build on studies which investigate the factors influencing language acquisition (Braginsky et al., 2019; Ghyselinck et al., 2004; Hills et al., 2010; Jones & Brandt, 2019; Peters & Borovsky, 2019; Roy et al., 2015; Sidhu et al., 2021), particularly on studies showing that sound symbolism helps language learning (Imai et al., 2008; Imai & Kita, 2014; Kantartzis et al., 2011) and that more systematic words are overrepresented among early acquired words (Monaghan et al., 2014). In detail, we investigate whether systematicity predicts AoA while controlling for neighbourhood density in both form and meaning, since recent studies have shown that words that are highly entrenched in the phonological and semantic networks are learned earlier (Fourtassi et al., 2020; Jones & Brandt, 2019). On the other hand, we leverage recently introduced measures of form-meaning systematicity which have been extensively validated on other psycholinguistic tasks such as lexical decision and reading (Amenta et al., submitted; Hendrix & Sun, 2020; Marelli & Amenta, 2018) and extend their use to the analysis of acquisition phenomena. These measures are theoretically relevant because they hold promise to characterise a mechanistic process that children could rely upon when exploiting systematicity in language learning.

## Materials and method

### Data

Several existing large-scale datasets were used to conduct this study. Objective AoA norms were taken from the dataset provided by Brysbaert and Biemiller (2017), which relies on vocabulary tests to assess when a given proportion of learners reliably know a certain word, probing learners at 2, 4, 6, 8, 10, 12, 13, and 14 years of age. This dataset provides different AoA estimates for different word senses of the same word form; however, since our approach does not discriminate word senses, we took the earliest AoA score for each word form as our target variable. Next to providing objective AoA norms for a large set of words, this dataset was also recently investigated by Sidhu and colleagues (2021) who reported an effect of iconicity on AoA. It is thus interesting to investigate the effect of systematicity on AoA on the same dataset, to better situate any finding in the current literature on non-arbitrariness and language learning.

Other large-scale datasets were used to derive control variables necessary to assess a possible unique effect of form-meaning systematicity on AoA. Concreteness norms were taken from the dataset collected by Brysbaert and colleagues (2014). Valence was extracted from the norms made available by Kuperman and colleagues (2014). Word frequency values come from the SUBTLEX-US dataset (Brysbaert & New, 2009). This dataset was also used as a reference vocabulary to retrieve phonological neighbours when computing PSC. However, we filtered the SUBTLEX-US lexicon by only considering words that appeared in at least one of the aforementioned datasets, to ensure that neighbours were valid English words, for a total of approximately 60 K words. To control for the morphological complexity of target words, a binary variable was created which marked words as either monomorphemic or polymorphemic: to this end, we used the MorphoLEX dataset (Sánchez-Gutiérrez et al., 2018). Iconicity ratings were taken from the dataset provided by Perry and colleagues (2015). To minimise the degrees of freedom in the computational modelling, we extracted a measure of PND from the MALD dataset (Tucker et al., 2019). The target variable we used is *PhonND* (PND), which reflects Coltheart’s *N*, i.e., the number of valid words that can be produced by changing a single phoneme in the target word (Coltheart et al., 1977).

Next to leveraging several datasets which provide lexical variables, we need resources to encode semantics, to compute SND and PSC measures. We chose to represent lexical semantics relying on distributed semantic representations extracted from large-scale corpora on the basis of the assumption that the meaning of a word can be approximated via its co-occurrence patterns with other words in the lexicon (Firth, 1957; Harris, 1954). In a distributional semantic model (DSM; Turney & Pantel, 2010), words are represented as numerical vectors obtained from co-occurrences in large text corpora. Crucially, these vectors exist in a geometrical space in which relations of proximity can be computed, with words with closer meaning being also closer in semantic space. Proximity is computed using the cosine of the angle between the vectors (Bullinaria & Levy, 2007). To derive lexical semantic representations, we relied on the DSM provided by Mandera and colleagues (2017), which has been validated on several psycholinguistic tasks.^2^ SND was quantified as the average cosine distance of the 20 nearest neighbours of a target word in semantic space; therefore, higher values on SND indicate that a word is found in a sparser semantic neighbourhood. Finally, to compute PSC, the phonological encoding of a word was extracted from the celex database (Baayen et al., 1996), while the corresponding lexical representation came from the same DSM used to compute SND: We describe the procedure to compute PSC in more detail in the following section. The final set of target words was determined by taking the intersection of the words in all relevant resources except for the iconicity norms, such that for each word all necessary values were available, resulting in 6,407 target words. Since iconicity norms are available for a considerably lower number of words, we ran separate analyses when we control for iconicity, targeting a smaller set of target words.

### Method

#### FSC

**Figure 1.**
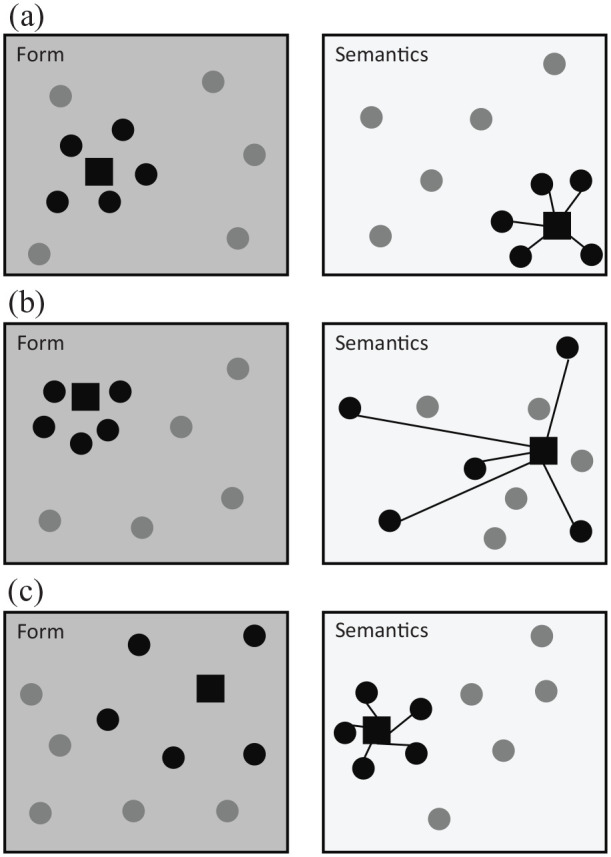
Schematic representation of Form-to-Semantics Consistency (FSC). (a) A word with high FSC and high neighbourhood density in form space: nearest neighbours in form space are also close neighbours of the target in semantic space, indicating a high consistency between the local neighbourhoods of the target word’s form and meaning. (b) A word with low FSC yet high neighbourhood density in form space: even though the target word occupies a dense portion of both form and meaning space, its nearest phonological neighbours are scattered in semantic space, resulting in low systematicity. (c) A word with high FSC in spite of low neighbourhood density in form space: even though the target word occupies a rather empty portion of form space, its nearest phonological neighbours cluster closely to the target in semantic space, showing that FSC and neighbourhood density in form space can be decoupled. Dark grey panels (left) represent form space as a geometrical, two-dimensional space where words are closer in space when their word forms are more similar. Light grey panels (right) represent semantic space also as a two-dimensional space, where points closer in space are semantically more similar. The black square indicates the target word for which FSC is being computed. Black circles indicate nearest neighbours to the target in form space. Dark grey circles represent other words in the vocabulary. FSC is computed by considering the dark segments which connect the target word (black square) to its form-based nearest neighbours (black circles) in semantic space. The lower the average length of the segments, the higher the FSC score for the target word.

Figure 1 provides a general graphical representation of how FSC measures are computed and how they tap into the interface between word forms and lexical semantics, by quantifying the degree of systematicity as the coherence of the semantic representations of the words which resemble the target in form space. The figure highlights how the measure is conceptualised and how its value depends on the coherence of local neighbourhoods in both form and semantic space, while remaining agnostic to the exact implementation choices about how to encode word form, how to retrieve nearest neighbours in form space, how to represent lexical semantics, and how to compute semantic similarity. As the figure shows, a word can have high FSC regardless of whether it has high density in form or semantic space: FSC will be higher when the nearest neighbours of a target word in form space are semantically coherent with the target word’s semantic representation. At a conceptual level, a high FSC score entails that upon hearing a certain word form, it is comparably easier to form a reliable impression of what this word may mean, even without relying on linguistic or situational context, but rather leveraging the meaning of the words that sound more like the target word itself. In the previous section, we established that word form is going to be represented using phonological transcriptions while semantic space is operationalised using DSMs. In the following paragraphs, we go into more details about how we implemented FSC in this work, explaining how we retrieve nearest neighbours in form space and how we compute the semantic similarity between their semantic representations and that of the target word.

As previously mentioned in the introduction, two operationalisations of FSC have been previously presented in the literature. These definitions differ in how the neighbours in form space are determined as well as in how the similarity between target and neighbours in semantic space is computed. In the original OSC measure introduced by Marelli and colleagues (2015), neighbours are defined as words that begin with the target string (e.g., *reduce* is a neighbour of *red* but *credit* is not). A later study by Marelli and Amenta (2018) considered various alternative definitions of OSC, reporting better results in predicting RTs in lexical decision when loosening the constraint that neighbours have to begin with the target and allowing for neighbours to simply embed the target (both *reduce* and *credit* are thus considered to be neighbours of *red* under this revised definition). For our analyses, which focus on phonology rather than orthography, we follow Marelli and Amenta (2018) and compute target-embedding PSC (*PSC_te_*) according to Equation 1:

(1) $$PSC_{te}(t)=\frac{\sum_{i=1}^{N}\cos(t,\;n_{i})\times f_{n_{i}}}{\sum_{i=1}^{N}f_{n_{i}}}.$$

*PSC_te_* for a target word *t* is thus computed by retrieving the semantic representation corresponding to the target word, *t*, and then computing the cosine similarity, cos(⋅,⋅) , to the semantic representation *n_i_* of each phonological neighbour *n_i_* out of the *N* words which embed the target. Cosine similarity is multiplied by the neighbour frequency, $f_{n_{i}}$ (retrieved from SUBTLEX-US), then each weighted cosine similarity is summed and the resulting value is normalised by the cumulative frequency of the neighbours. Therefore, more frequent neighbours contribute more to the final PSC value. It is of course possible that a word’s phonological form is not embedded in any other word; in this case, we randomly sampled 20 words as the target’s form-based neighbours and proceeded with the computation as detailed in Equation 1.

As previously discussed in the introduction, Hendrix and Sun (2020) computed OSC in a different way, which had also been tested by Marelli and Amenta (2018) but was found to be worse at predicting RTs in lexical decision. In this approach, form-based neighbours are words with the smallest Levenshtein distance to the target. Moreover, instead of weighing cosine similarities by word frequency, these are weighted by the Levenshtein distance value, such that semantic representations corresponding to orthographically more similar words contribute more to the OSC estimate. This implementation of OSC has one free parameter, *k*, i.e., the number of form-based neighbours to consider: This parameter was set to five, following Hendrix and Sun (2020).^3^ However, it can easily be the case that several words have the same Levenshtein distance to the target and that these words are more than is specified by *k*. Hendrix and Sun (2020) solved this issue by sampling *k* words out of those at the same distance from the target. However, we take a different approach. Consider the word *water* with orthographic neighbours and Levenshtein distance (indicated in parentheses) *eater* (1), *later* (1), *waiter* (1), *gate* (2), *whiter* (2), *laser* (2), etc. The fifth closest neighbour is at a distance of two, but there are more at the same distance (and which one appears as the fifth depends on alphabetical order or randomness); therefore, we take the distance of the fifth closest neighbour and consider as nearest neighbours of the current target all words with a distance lower than or equal to that of the fifth neighbour itself. In the example case, all words with a Levenshtein distance of one or two would count as form-based neighbours of *water*, and their semantic representations would be used to compute OSC. See Equation 2 for how we implemented Levenshtein-distance PSC (*PSC_ld_*), which mimics OSC but works off phonological rather than orthographic representations.

(2) $$PSC_{ld}(t)=\frac{\sum_{i=1}^{N}\cos(t,n_{i})\times 1/d_{i}}{N}.$$

*PSC_ld_* for target word *t* is computed similarly to *PSC_te_: t* is the target word’s semantic representation, *n*_i_ indicates each of the *N* with phonological neighbours of the target word and *n_i_* is the corresponding semantic representation, *d_i_* is the Levenshtein distance between the phonological form of the target word *t* and the phonological form of neighbour *n_i_*. Finally, cos(⋅,⋅) indicates the cosine similarity in semantic space.

### Statistical approach

All statistical analyses were carried out in R (R Core Team, 2020). First, we applied a Box-Cox transformation to all numerical independent variables using the MASS package (Venables & Ripley, 1999) and then *z*-standardised them. Then, we carried out a correlational analysis to characterise the relations between the different variables and in particular how PSC measures relate to AoA.

Moreover, to assess whether PSC explains any unique variance in AoA on top of known predictors, we ran several linear regression models. First, we fit a baseline statistical model where AoA is modelled as a linear combination of frequency, concreteness, word length in phonemes, valence, morphological complexity, PND, and SND. We then added target predictors and interactions to this baseline statistical model, measuring the difference in Akaike Information Criterion (AIC) between different models to assess whether adding a certain predictor indeed improved model fit.

Next to this main analysis, we also controlled for the effect of iconicity. To do this, we restricted our focus to the words for which all necessary ratings are available, resulting in 1,771 words. First, we fitted a baseline statistical model which included the same predictors as the main baseline plus iconicity ratings. We then added each PSC measure individually and measured the ∆_*AIC*_.

In both analyses, there is a risk of observing adverse effects of collinearity (Tomaschek et al., 2018). To ensure any reported effect is robust and trustworthy, we ran the same models using Random Forest (RF) regression using the *ranger* package (Wright & Ziegler, 2017). RF regression is a machine learning method based on decision trees and recursive partitioning. The trees are fit to a subset of the data and only use a subset of the predictors (a third of the available variables in our analyses). The predictions of many hundreds of trees are then averaged, which helps avoid overfitting and increases accuracy. RF regression does not suffer from collinearity as it takes random subsets of the independent variables for every tree it builds and does not need to estimate a coefficient for the independent variables (Tomaschek et al., 2018). Rather, the relative importance of two highly correlated predictors can be compared by assessing the trees in which they do not co-occur. Finally, the importance of a variable to the regression task can be easily computed and provides insights about its role in predicting the target variable. All of the datasets and code to replicate the analyses presented here can be found online at https://github.com/niklim/FSC_and_AoA.

## Results

The results are organised in four different subsections. First, we discuss the correlational analysis, focusing on the relation that PSC has with AoA as well as with other variables known to influence AoA. Second, we assess the unique effect of PSC on AoA after factoring in known predictors of AoA. Third, we assess whether any effect of PSC can be reduced to an effect of iconicity (Dingemanse et al., 2015; Perry et al., 2015). Finally, we zoom in on the relation between PSC and SND, following the hypothesis that sparser semantic neighbourhoods can afford more non-arbitrary relations between form and meaning (Gasser, 2004; Sidhu & Pexman, 2018b) and to test whether this relation influences AoA.

### Correlational analysis

**Figure 2.**
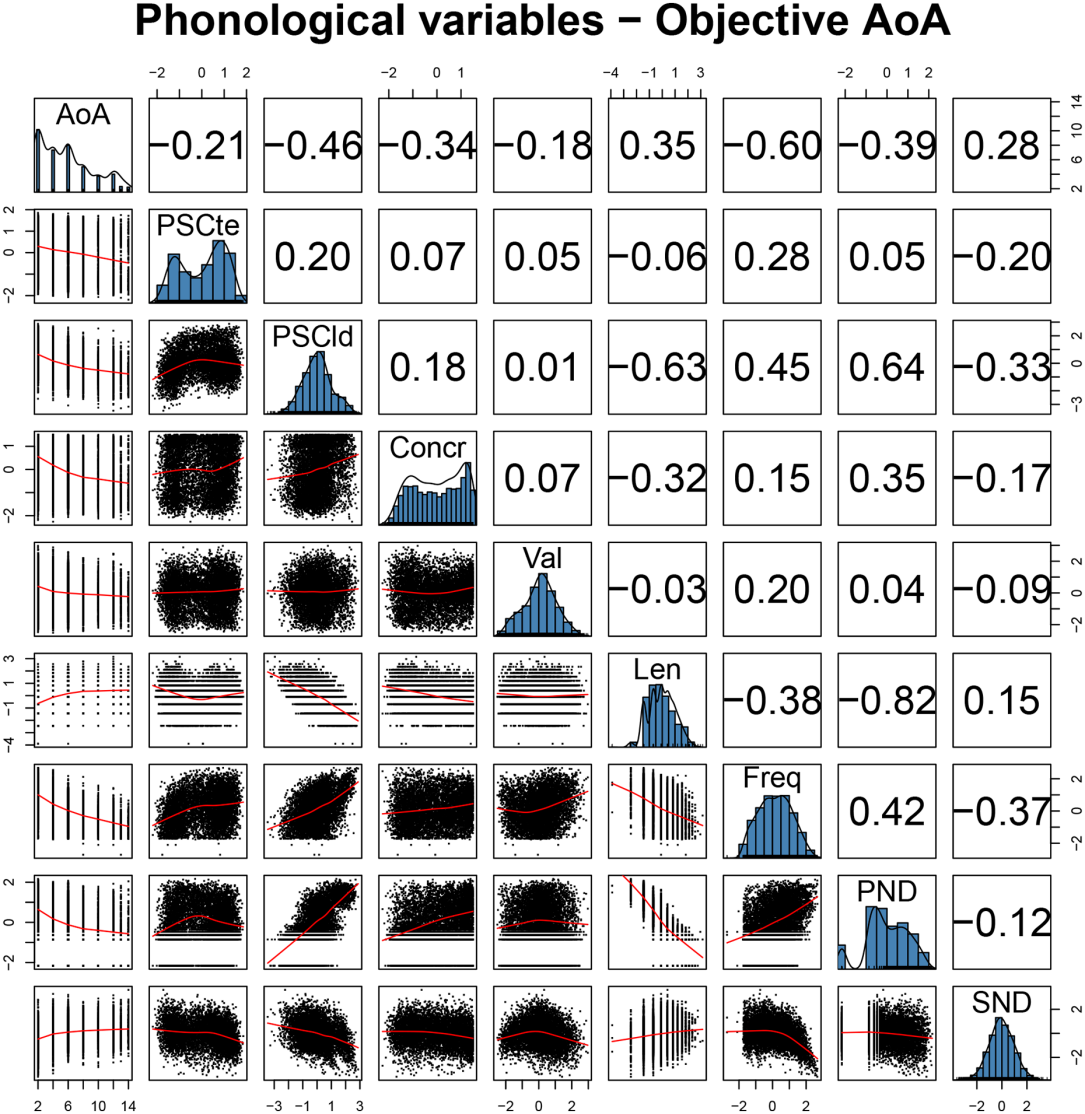
Pairwise Pearson’s correlations involving independent variables and AoA norms for phonological variables. The main diagonal provides histograms showing the distribution of each variable; the upper triangle provides correlation coefficients, the lower triangle provides scatterplots, where the red line is a LOESS fit. AoA: Age of Acquisition; Freq: frequency; Concr: concreteness; Val: valence; SND: Semantic Neighbourhood Distance; Len: length in phonemes; PND: Phonological Neighbourhood Density.

First, we computed pairwise Pearson’s correlation coefficients for all variables, displayed in Figure 2, together with histograms showing the distribution of each variable and scatterplots showing how variables relate to each other. We see that AoA norms have sizable correlations with all variables, including the expected negative correlations with PSC measures, indicating that words with higher systematicity tend to be acquired earlier. However, the correlation between *PSC_te_* and AoA (*r* = −.21) appears to be considerably smaller than the correlation between *PSC_ld_* and AoA (*r* = −.46).

We also observe the expected relation between AoA and PND (*r* = −.39). Since Coltheart’s *N* is higher when there are more neighbours that can be obtained by applying a single transformation to the target word, high values on PND indicate denser phonological neighbourhoods, and the negative correlation shows that words tend to be acquired earlier when they are found in denser phonological neighbourhoods. Finally, the relation between AoA and SND is also in the expected direction (*r* = .28), with words found in denser semantic neighbourhoods being learned earlier. As previously mentioned, SND is a measure of distance; hence, it is higher when neighbours are found further from the target, and the target is thus in a sparser semantic neighbourhood.

Finally, we observe collinearity across predictor variables, especially involving word length in phonemes and PND (*r* = −.82), which can cause brittleness in the estimation and warrant the use of RF regression to check the robustness of any reported pattern. Another interesting observation is that *PSC_te_* and *PSC_ld_* seem to tap into different aspects of form-meaning systematicity, with a low to moderate pairwise correlation (*r* = .2).

### Form-meaning systematicity and AoA

In this section, we assess whether PSC measures explain any unique variance in AoA once control variables are accounted for: This analysis is particularly relevant considering that especially *PSC_ld_* has sizable correlations with frequency, length in phonemes, PND, and SND, raising the doubt that its correlation with AoA can be reduced to the effects of other lexical variables. We started by fitting a baseline linear regression model, $aoa_{base}$ , for predicting AoA norms using only the control variables. $aoa_{base}$ had an AIC of 30,442.48, and all independent variables reliably predict AoA norms (all *p* < .001).

Then, we compared $aoa_{base}$ to a regression model including the same predictors as well as an interaction term involving PND and SND, ath $aoa_{pnd*snd}$ , which had an AIC of 30,443.12 (∆_*AIC*_ = −0.639), with the interaction term failing to reach significance (β = .039, *se =* 0.033, *t* = −1.166, *p* = 0.244). It does not seem to be the case, then, that word learning is affected by an interaction of PND and SND. If we were to find an effect of PSC on AoA, therefore, it could be entirely ascribed to an effect of systematicity rather than to the concurrent entrenchment of words in the phonological and semantic networks.

We thus proceeded to fit two further models: adding *PSC_te_* and *PSC_ld_* to the predictors in $aoa_{base}$ , since the interaction between PND and SND did not improve model fit. Results are summarised in Table 1: ∆_*AIC*_ scores are computed relative to $aoa_{base}$ .

**Table 1.** Unique effect of PSC on objective AoA norms.

Measure	β	*SE*	*t*	*p*	∆_*AIC*_
*PSC_te_*	−0.145	0.034	−4.263	<.001	16.17
*PSC_ld_*	−0.738	0.046	−15.968	<.001	248.38

PSC: Phonology-to-Semantics-Consistency; AIC: Akaike information criterion.

The table displays regression coefficients (β) with associated standard errors (*SE*), *t* statistics, *p* values and difference in AIC with respect to the corresponding baseline statistical model (∆_*AIC*_).

PSC measures explain unique variance in AoA on top of control variables, suggesting that form-meaning systematicity has a unique relation with acquisition patterns. *PSC_ld_* brought a larger improvement in model fit (∆_*AIC*_ = 248.38) than *PSC_te_* (∆_*AIC*_ = 16.17). Both measures, nonetheless, proved to reliably predict AoA in the hypothesised direction, with more systematic words being learned earlier (*PSC_te_*: β = −.145, *SE* = 0.034, *t* = −4.263, *p* < .001; *PSC_ld_*: β = −.738, *SE =* 0.046, *t* = −15.968, *p* < .001).

**Figure 3.**
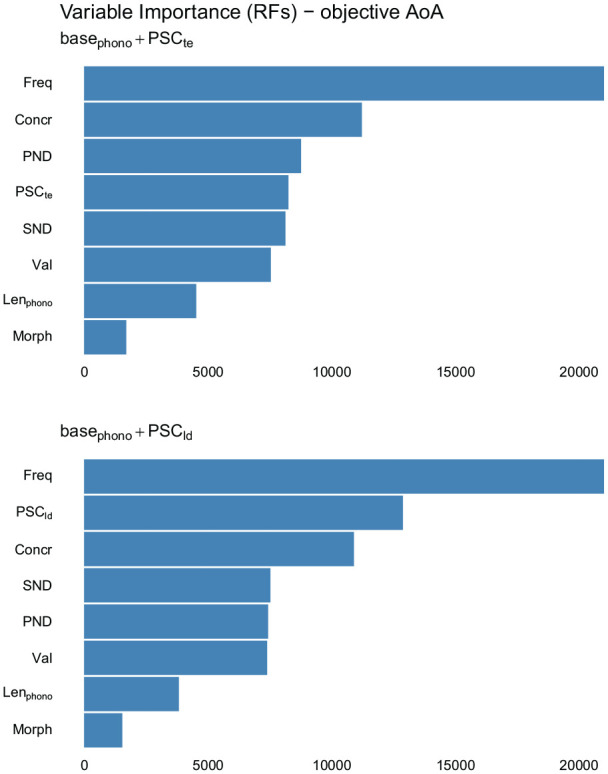
Variable importance plots from RF regressions for models including measures of form-meaning systematicity on top of the baseline statistical model predicting objective AoA norms. RF: Random Forest; AoA: Age of Acquisition; *PSC_ld_*: Levenshtein-distance Phonology-to-Semantics-Consistency; *PSC_te_*: target-embedding Phonology-to-Semantics-Consistency; Freq: frequency; Concr: concreteness; Val: valence; SND: Semantic Neighbourhood Distance; Len_phono_: length in phonemes; PND: Phonological Neighbourhood Density; Morph: morphological complexity (binary).

The RF regressions confirmed that all PSC measures improve the model’s *r*^2^: The improvement brought by *PSC_te_* is 0.035, while that brought by *PSC_ld_* is of 0.057. Plots showing variable importance, provided in Figure 3, confirm that FSC measures contribute substantially to the prediction of AoA. *PSC_ld_*, in particular, is slightly more important than concreteness and more important than most other predictors. These analyses thus confirm that PSC has a reliable unique relation with AoA, such that words with a higher degree of form-meaning systematicity tend to be learned earlier.

### Form-meaning systematicity, iconicity, or both?

In this section, we focus on a further possible confound which was not addressed in the previous analysis, namely iconicity. We chose to present separate analyses because iconicity ratings are available for a considerably smaller subset of the target vocabulary. After the previous analyses, we can say that PSC measures have a unique effect on AoA. If we were not to find the same effect we have encountered so far after controlling for iconicity, we would be more confident that it is because PSC and iconicity tap into similar aspects of the acquisition processes and its relation to lexical properties. Since it came out as a more reliable and better predictor of AoA than *PSC_te_*, we only consider *PSC_ld_* in the following analyses.

First of all, we checked to what extent *PSC_ld_* correlates with iconicity and to what extent iconicity correlates with AoA norms. For each correlation coefficient, we provide the point estimate as well as the 95% confidence interval (CI) in square brackets, the *t* statistic, the degrees of freedom (*df*), and the *p* value. We observe small albeit reliable correlations between AoA and iconicity (*r* = −.059, 95% CI = [−0.106, −0.013], *t* = −2.503, *df* = 1,769, *p* = 0.012). Iconicity also correlates positively with *PSC_ld_* (*r* = 0.161, 95% CI = [0.115, 0.206], *t* = 6.865, *df* = 1,769, *p* < .001). Therefore, it is possible that iconicity explains variance in AoA that *PSC_ld_* also accounts for.

**Figure 4.**
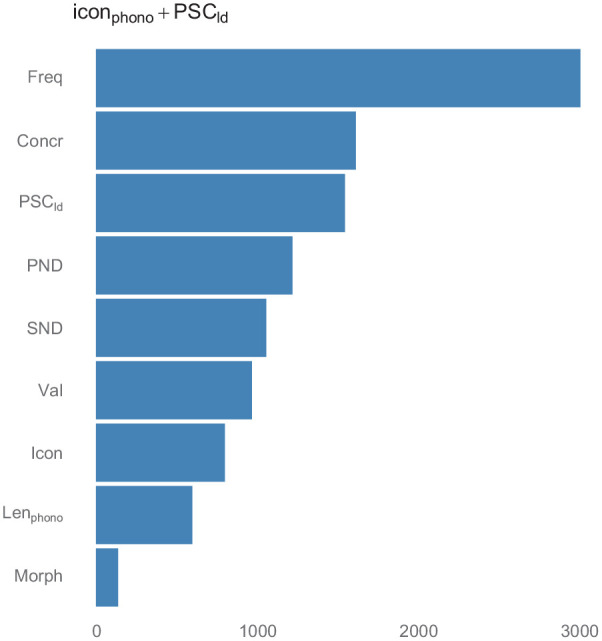
Variable importance plots from RF regressions for models including *PSC_ld_* on top of the baseline statistical model including iconicity. RF: Random Forest; *PSC_ld_*: Levenshtein-distance Phonology-to-Semantics-Consistency; Freq: frequency; Concr: concreteness; Val: valence; SND: Semantic Neighbourhood Distance; Len_phono_: length in phonemes; PND: Phonological Neighbourhood Density; Morph: morphological complexity (binary); Icon: iconicity.

Once again, we fitted a baseline regression model, $aoa_{icon}$ , in which AoA norms are modelled using the predictors from $aoa_{base}$ plus iconicity, which had a reliable effect (β = −.349, *SE* = 0.046, *t* = −7.656, *p* < .001). Then, we added *PSC_ld_* to $aoa_{icon}$ and measured the ∆_*AIC*_: the target variable improved model fit (∆*_AIC_ =* 16.44) and had a reliable negative effect on AoA (β = −.295, *SE* = 0.069, *t* = −4.293, *p* < .001). Therefore, the effect of *PSC_ld_* on AoA appears to be robust also after controlling for iconicity (which remains a reliable predictor). The pattern reported for the linear regression is largely confirmed by the RF regression: *PSC_ld_* improves the model’s *r*^2^ by 0.053 points. Figure 4 displays variable importance scores, showing that *PSC_ld_* is more important than iconicity in predicting AoA and ranks just below concreteness.

To sum up, we confirmed a relation between *PSC_ld_* and AoA even after controlling for iconicity, and showed that it has a sizable effect on AoA, which is particularly evident when considering the importance of *PSC_ld_* in RF regression.

### Form-meaning systematicity and SND

In the previous analyses, we established that *PSC_ld_* has a unique and reliable relation with AoA, which holds after controlling for iconicity. In this section, we investigate whether, following Gasser (2004), words in sparser semantic neighbourhoods have higher PSC, and further investigate whether this relation explains unique variance in AoA.

We start by observing that *PSC_ld_* has a small to moderate negative correlation with SND (*r* = −.33, see Figure 2), indicating that more systematic words tend to be found in denser semantic neighbourhoods, contrary to what we hypothesised based on the theory by Gasser (2004). The same relation emerged when fitting a linear model predicting *PSC_ld_* using SND while controlling for word length in phonemes, PND, frequency, and AoA in line with the approach taken by Sidhu and Pexman (2018b). The β coefficient of SND was reliably different from 0 and negative, indicating that when semantic density decreases (so SND increases), systematicity decreases (β = −.183, *SE* = 0.009, *t* = −19.275, *p* < .001). It seems therefore to be the case that words in sparser semantic neighbourhoods tend to show less consistent form-meaning mappings, unlike what Sidhu and Pexman (2018b) reported about the relation between SND and iconicity, which we replicate (*r =* .064, 95% CI = [0.017, 0.110], *t* = 2.688, *df* = 1,769, *p* < .01). The next question to be answered is whether this relation between SND and PSC bears relevance to acquisition patterns.

**Figure 5.**
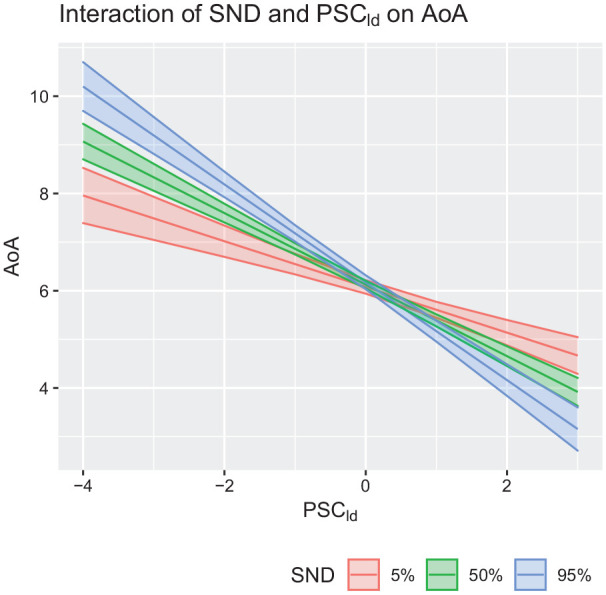
Interaction between Semantic Neighbourhood Distance (SND) and *PSC_ld_* on objective AoA norms. The colour legend should be interpreted as follows: 5% indicates words found in dense semantic neighbourhoods; 50% indicates words found in semantic neighbourhoods of average density; 95% indicates words found in sparse semantic neighbourhoods. *PSC_ld_*: Levenshtein-distance Phonology-to-Semantics-Consistency; AoA: Age of Acquisition.

We again fitted a linear regression model adding an interaction between SND and *PSC_ld_* to $aoa_{base}$ . Adding the target interaction did improve the model fit (∆_*AIC*_ = 30.33), with a significant interaction coefficient (β = .165, SE = 0.029, *t* = 5.689, *p* < .001). Figure 5 provides a graphical representation of the interaction, where we see that the negative effect of *PSC_ld_* on AoA norms is stronger for words in sparser semantic neighbourhoods. This pattern is in line with the prediction by Gasser (2004) and the empirical evidence provided by Sidhu and Pexman (2018b).

Therefore, we only partially replicated previous findings, which reported a robust relation between non-arbitrary form-meaning mappings and SND. On one hand, we did see a positive correlation between iconicity and SND, indicating that more iconic words tend to exhibit lower semantic density, in line with evidence from Sidhu and Pexman (2018b). On the other hand, however, we saw a relation in the opposite direction than we predicted (Gasser, 2004) between *PSC_ld_* and SND, with words in sparser semantic neighbourhoods showing less, not more, systematicity. Finally, we observed that an interaction between SND and *PSC_ld_* explained additional variance in acquisition patterns, above and beyond that explained by control variables. In detail, the negative effect of *PSC_ld_* on AoA was stronger for words in sparser semantic neighbourhoods, suggesting that learners may benefit more from systematicity when words are found in sparser portions of semantic space, offering another source of information during word learning. This observation fits with the predicted relation between systematicity and SND, but given the contradicting findings, these results should not be over interpreted.

### Robustness checks

We further carried out four different robustness checks (whose details and outcomes are available as online supplementary materials) to ensure that the reported unique relation between PSC and AoA is reliable. The first one was meant to ensure that the effects we reported do not depend on the assumption of a linear relation between our predictors and AoA. For this analysis, we used Generalised Additive Models (GAMs, Wood, 2017) to predict AoA, modelling each predictor as a simple smooth. We first fitted a baseline statistical model including control variables, then tested whether an interaction between PND and SND (implemented as a partial tensor product) improved the model fit by checking the ∆_*AIC*_. Then, we separately added each PSC measure to the baseline statistical model, again measuring the ∆_*AIC*_ to check whether they further improved the model fit. The most important patterns were replicated, with *PSC_ld_* improving the model fit more than *PSC_te_* and showing a largely linear relation with AoA. More details are provided in Supplemental Appendix A.

The second robustness check tested whether the effect of PSC truly depends on systematic form-meaning correspondences in the lexicon and is not a by-product of other properties of the semantic representations. To probe this, we scrambled the form-meaning correspondences, such that, for example, the word form *cat* no longer necessarily corresponds to the semantic representation for cat. This random permutation ensures that neighbourhood relations in phonological space and semantic space remain intact (the number and distance of nearest neighbours is the same), only affecting the way in which form and meaning interact (neighbouring representations point to different lexical items). We randomly permuted the semantic space 1,000 times, and each time derived PSC measures from random form-meaning correspondences, measuring the pairwise correlation between PSC and AoA, and the ∆_*AIC*_ between the baseline regression model, $aoa_{base}$ , and the models including random PSC measures, deriving a distribution under random permutations. Surprisingly, *PSC_ld_* had a reliable non-zero correlation with AoA, with the distribution centred on −0.25. Nonetheless, once random PSC measures were added to the baseline statistical model, they did not improve model fit, confirming that the unique relation between PSC and AoA we documented in the main analysis is only found when leveraging true form-meaning correspondences in the English lexicon. Further details about this analysis are provided in Supplemental Appendix B.

The third robustness check considers the role of the reference lexicon used to retrieve nearest neighbours when computing PSC, to exclude the possibility that the relation we showed is only found with a large or specific reference lexicon. To this end, we randomly sampled 50% or 75% of the words in the reference lexicon without replacement. For each sampling rate, we carried out 500 iterations; in each of them, we retrieved phonological nearest neighbours from the vocabulary subset, and computed PSC measures as detailed in Equations 1 and 2. We then measured the ∆_*AIC*_ between $aoa_{base}$ and the regression models including PSC measures. Results show that PSC measures improve model fit also when computed on vocabulary subsets. More details can be found in Supplemental Appendix C.

The fourth robustness check we carried out was meant to further ensure that the effect of PSC is not an epiphenomenon of simpler neighbourhood measures. Even though the main analysis explicitly controls for PND and SND, and although the random baseline confirms that, even though PSC computed from random permutations of form-meaning correspondences correlates with AoA, it does not explain any unique variance after control variables are considered, we ran an extra analysis which more carefully disentangles the effects of PND, SND, and *PSC_ld_* on AoA. We focused here only on *PSC_ld_* since it has proven to be a better predictor of AoA throughout all other analyses and robustness checks. We started by running a Principal Component Analysis (PCA) on PND, SND, and *PSC_ld_*, deriving three orthogonal principal components (PCs) that account for all the variance in the original variables but exclude collinearity. We then ran a linear regression where the three PCs were added to the other control variables. We found that the third PC reliably and positively predicts AoA. Importantly, the third PC correlates positively with PND but negatively with *PSC_ld_*, such that words with high values on the third PC tend to have lower systematicity yet higher PND. Crucially, the positive effect of the third PC on AoA aligns with the predicted effect of *PSC_ld_* on AoA but contradicts that of PND, confirming that our measure of form-meaning systematicity relates to AoA beyond, and differently from, PND. Further details are provided in Supplemental Appendix D.

Finally, we also replicated the analysis presented in the main text by replacing PSC with OSC, to ensure that patterns are reliable when changing the words’ encoding (Supplemental Appendix E) and also by predicting subjective rather than objective AoA norms (Kuperman et al., 2012), as reported in Supplemental Appendix F. All patterns we found for PSC are also reported for OSC, with some differences in magnitude pointing to a stronger effect for form-meaning systematicity at the phonological level. This is interesting since language learning, especially early on, relies a lot more on spoken rather than written input. Patterns were also largely replicated when predicting subjective AoA norms, although some differences emerged. First, the interaction between SND and PND was significant, with a stronger negative effect of PND on AoA for words in dense semantic neighbourhoods. In line with the analysis on objective AoA norms, however, the main effects of *PSC_te_* and *PSC_ld_* were significant, both in the general analysis and after controlling for iconicity. Unlike what was reported for objective AoA norms, though, the interaction between SND and *PSC_ld_* did not have a reliable effect on AoA.

## Discussion

In this article, we investigated the effect of form-meaning systematicity (Amenta et al., 2017; Hendrix & Sun, 2020; Marelli & Amenta, 2018; Marelli et al., 2015) on word acquisition. We first documented a reliable correlation between Phonology-to-Semantics Consistency (PSC) and AoA. Then, we showed that this relation is not confounded by other known predictors of AoA such as frequency, concreteness, valence, word length, and morphological complexity. Crucially, we further showed that the effect of PSC holds when PND and SND are controlled for. Therefore, we showed that the effect of PSC on AoA is not just due to words being entrenched in the phonological and semantic networks but depends on form-meaning systematicity. Moreover, we showed the robustness of the reported patterns by replicating the analyses using RF regressions, which remove possible adverse effects of collinearity (Tomaschek et al., 2018). Finally, we showed that the relation between systematicity and AoA is robust to the inclusion of iconicity (Perry et al., 2015; Sidhu et al., 2021), suggesting that systematicity and iconicity tap into different aspects of acquisition dynamics.

The effect of form-meaning systematicity on AoA proved reliable also in a series of robustness checks. We made sure that the target variables were reliable predictors of AoA also when relaxing the assumption of a linear relation between the variables, using GAMs. We further tested a random baseline where form-meaning correspondences found in the English lexicon were randomly scrambled, showing that PSC only predicts AoA when computed on true form-meaning pairings. We also ensured that the effects we report are robust to changes in the size and composition of the reference vocabulary used to retrieve nearest neighbours when computing PSC. Moreover, we ran an extra analysis to disentangle the effects of PSC, PND, and SND, showing that the effect of PSC on AoA is not an epiphenomenon of neighbourhood density in form or meaning. We further controlled for changes in the encoding of word form (orthography and phonology) and probed subjective AoA norms. Across all these analyses, the main effect of systematicity proved robust. However, target interactions between PND and SND, and between SND and FSC did not; they were reliable predictors in some analyses while they did not improve the model fit in others, inviting caution in drawing firm conclusions about these patterns.

Our work builds on recent studies that quantified form-meaning systematicity above and beyond morphological productivity. The main advantage of FSC measures over alternative approaches to computing form-meaning systematicity (Dautriche et al., 2017; Monaghan et al., 2014; Pimentel et al., 2019; Shillcock et al., 2001; Tamariz, 2008) is that FSC measures work even when a word’s semantic representation is not available (Hendrix & Sun, 2020). This makes FSC more appealing to explore acquisition dynamics since a hallmark of form-meaning systematicity is the possibility of drawing on statistical regularities between form and meaning available in the lexicon when learning and processing language (Monaghan et al., 2012). Therefore, FSC measures can characterise how statistical regularities between form and meaning help children extrapolate meaning from form alone and use these informed guesses to better learn new words. While FSC measures have so far been used simply to quantify the degree of form-meaning consistency, it is rather straightforward to construe them as fully fledged mapping functions; the semantic vectors of the form-based neighbours could be averaged to obtain a new semantic representation, which would exist in the same semantic space derived from contextual information (Firth, 1957; Harris, 1954) and which would conflate the semantics of the most similar words in the vocabulary.

We can sketch a possible mechanistic process that relies on the principles upon which FSC is based to see the possible role of systematicity in word learning. Let us suppose that a learner encounters a novel word for the first time, e.g., *mordor*. Let us further assume that this first encounter happens in a limited linguistic and situational context, e.g., *Tomorrow we go to the mordor*, such that it is harder for the learner to quickly pin down the semantics of this novel word. Given the sentence in which it appears, the *mordor* is likely a place, but the linguistic context does not afford more precise semantic inferences about it. If, however, the form of this word reliably cues its semantics, the learner can sidestep the limited context and derive a more precise characterisation of the novel word’s semantics based on its form. For example, a learner could leverage the phonological similarity between *mordor* and words such as *sordor* or *murder*, combine their semantics, and infer that going to the *mordor* may not be the prettiest experience. The pseudoword *mordor* would thus have a high FSC, since the closest phonological neighbours have a rather coherent semantics, and is predicted to be easier to learn based on our results. On the contrary, a pseudoword such as *dord*, with semantically different neighbours like *cord*, *bord*, *ford*, *dorm*, *lord*, would have a low FSC, and make it harder to infer its semantic connotations without an informative linguistic or situational context.

The process by which one infers a novel word’s semantics leveraging the semantics of similar words, however, can only work if the semantic intuitions derived in this way prove to be generally correct, such that later encounters with the same word, in richer situational and linguistic contexts, do not disprove the form-based semantic intuitions. In other words, this process can only work if the lexicon exhibits form-meaning systematicity in the early stages of word learning. Our results show precisely this, i.e., that English words for which it is easier to derive coherent semantic impressions from similar words tend to be learned earlier, suggesting a place in word learning for an analogical process which bridges word form and meaning. Importantly, we do not contend that this process is always correct, such that the semantics inferred on the basis of word form reflects the true lexical semantics of every word, but rather that words whose phonological neighbours have more coherent semantics are easier to learn.

Therefore, the role of systematicity in language learning is taken to be different from what has been hypothesised in previous studies which target language processing. When considering lexical decision, the influence of FSC on RTs has been ascribed to a stronger activation of lexical items which display higher systematicity, which depends on the concurrent activation of coherent local neighbourhoods in form and meaning. Moreover, when considering reading times, it has been shown that FSC is particularly useful when the context is not particularly constraining, with form-based semantics offering an extra cue and filling in the gap left by an unconstraining linguistic context. When it comes to language learning, however, we hypothesise that the effect of systematicity works by offering a way to more quickly establish semantic representations by combining contextual information with information coming from the word form itself. Words for which this form-meaning systematicity does not exist would thus be harder to learn, since learners need to resolve a conflict, where form-based semantics does not align with contextual semantics. Whereas learners likely end up resolving this conflict by primarily relying upon contextual information, they may still have to understand which source of information is more reliable and find it easier to learn words for which these two sources of information agree. Furthermore, form-based semantic intuitions may be particularly useful with novel words, where contextual information may be scarce or unreliable.

If the process we have just outlined works, and the resulting semantic intuitions are consistent with the true lexical semantics of the words being learned, children could thus considerably speed up word learning by bootstrapping lexical learning using word forms and the semantics they convey. Under a full arbitrariness of the sign hypothesis, this process would be irrelevant since no useful semantic information could be derived from word form, except for morphological compositionality. However, we showed that PSC reliably and uniquely explains variance in acquisition patterns, with more systematic words being learned earlier. Therefore, our study provides preliminary evidence that words have a learning advantage when the form-based semantic inference reflects the lexical semantic representation derived from linguistic context. We can thus hypothesise that the exemplar-based process at the heart of FSC at least partly accounts for lexical acquisition. Importantly, this is the first work to show that a measure of form-meaning systematicity which can model how semantics is extracted from word form alone reliably predicts AoA patterns for large-scale datasets.

Through a number of analyses and robustness checks, we further showed that target-embedding FSC measures explain less variance in AoA than Levenshtein-distance FSC measures. This result contrasts with evidence from Marelli and Amenta (2018) about lexical decision data, where target-embedding measures were better. We suggest that this dissociation is theoretically relevant. Target-embedded neighbours are by necessity longer than the target itself. Thus, to estimate the degree of form-meaning systematicity for a shorter word, the learner should already know longer words than the target. While tenable when dealing with adult-sized lexicons, this assumption contrasts with developmental trajectories, where shorter words are learned earlier on average. On the contrary, Levenshtein-distance FSC does not depend on longer words being known and actually favours words with comparable lengths, offering a more plausible account of how similarity in form space is computed during learning. Thus, if the lexicon offers systematicity at a more coarse level, such that looser similarities in word form that can be leveraged when retrieving form-based nearest neighbours based on Levenshtein distance already cue something about word meaning, then learners can exploit word forms to infer semantics. Our results favour this interpretation.

Moreover, when reviewing the results of the correlational analysis, we noted how the two implementations of FSC correlated only moderately, suggesting they pick up on different aspects of form-meaning systematicity. It is however still interesting to note the predicted relation between AoA and target-embedding FSC, since it highlights a structural property of the lexicon. Target-embedding FSC measures may thus not characterise a mechanistic process by which learners infer novel words’ semantics based on phonological similarities, but still highlight that words for which phonological neighbours are semantically more coherent tend to be learned earlier. At the same time, however, Levenshtein-distance FSC measures may be too unconstrained, since neighbours may be retrieved for any string, phonotactically legitimate or not. Recently, Delgado and colleagues (2020) have provided preliminary evidence that sound-symbolic effects may depend on the degree to which non-words are acceptable in terms of phonotactics. Hence, future work should investigate and contrast different ways of deriving FSC measures, with a focus on which method best characterises how learners analogise over word forms during development.

The conceptualisation we have presented of FSC as a mapping function, which projects novel words onto semantic space, is in line other recent attempts to explicitly model a mapping function from word form to lexical meaning (Baayen et al., 2019; Cassani et al., 2020; Chuang et al., 2021; Hendrix & Sun, 2020). Hendrix and Sun (2020) relied on FastText (Bojanowski et al., 2017), a generalisation of word2vec (Mikolov et al., 2013) where semantic vectors for words are built by combining semantic vectors of character n-grams. FastText was introduced to improve the quality of semantic vectors for morphologically rich languages, where the very low chance of observing certain inflected forms in a corpus jeopardises the quality of the corresponding word vectors. By leveraging the systematicity inherent in morphological productivity, FastText derives semantic vectors for part-words from all the occurrences of each n-gram, leveraging contextual information. If a new, inflected form is found, the corresponding semantic vector will thus combine the semantics of all the n-grams it consists of, offering a better representation than would be obtained by simply leveraging the limited linguistic context in which the rare inflected form was found in the corpus. This approach can however generalise to entirely new words and illuminate how semantic intuitions can be derived for unknown words even when they offer no obvious morphological structure. The functioning of FastText suggests another venue for future research, namely investigating to what extent frequency and FSC are related. While it is easier to establish the semantic content of a word from context if the word occurs often, this gets harder the rarer a word is; form-meaning systematicity may thus be more important to learn and process infrequent words.

A different approach to map form and meaning (Baayen et al., 2019; Cassani et al., 2020; Chuang et al., 2021) uses simple linear mappings to learn a function which takes a form-based representation and projects it onto the same semantic space as context-based semantic vectors, not unlike other approaches in the study of morphology (Marelli & Baroni, 2015; Marelli et al., 2017). In this line of work, word form has so far been encoded using orthographic or phonological n-grams as well as audio features, whereas semantic representations have typically consisted of sparse distributed vectors derived from linguistic context using error-driven learning (Rescorla & Wagner, 1972). These measures have been primarily used to investigate the influence of form-meaning mappings in lexical decision tasks, both visual and auditory, with only a small-scale study that targeted acquisition phenomena (Cassani et al., 2020). In this work, semantic vectors derived from pseudowords were analysed to show that they entertain predictable relations with other semantic representations at the level of lexical categories (Monaghan et al., 2012), suggesting that a direct form-meaning mapping could help children form expectations about the syntactic role as well as the possible referent of a novel word (Fitneva et al., 2009). However, whereas these approaches seek to find an optimal solution to form-meaning mappings for the entire lexicon, FSC measures only target systematic relations within local neighbourhoods in form and meaning and rely on an analogical principle. Future work should focus on whether the algorithmic differences underlying different approaches to (and measures of) form-meaning systematicity bear relevance to psycholinguistic phenomena.

Next to discussing how the measures we tested can capture mechanisms relevant to word learning, it is important to discuss in more detail why form-meaning systematicity might help acquisition in the first place. We started by considering evidence that words in denser semantic as well as phonological neighbourhoods tend to be learned earlier (Fourtassi et al., 2020; Jones & Brandt, 2019). This suggests that words which are more entrenched in the language network at the level of form and meaning are easier to learn, in line with evidence by Hills and colleagues (2009) that the structure of the language network itself drives acquisition. Our analyses however show that the effect of form-meaning systematicity on AoA cannot be reduced to neighbourhood density in form and meaning. What could the advantage that form-meaning systematicity brings? Several studies have documented that children learn more iconic and systematic words earlier and more easily (Brand et al., 2018; Imai et al., 2008; Imai & Kita, 2014; Kantartzis et al., 2011, 2019; Laing, 2019; Lockwood et al., 2016; Maurer et al., 2006; Monaghan et al., 2011, 2012; Motamedi et al., 2020; Nygaard et al., 2009; Perniss & Vigliocco, 2014), with one study also showing that children are particularly sensitive to sound-symbolic patterns in language EEG (Kovic et al., 2010). This evidence aligns with the theory by Gasser (2004) that systematicity helps learning by making it possible to leverage analogies in form to infer something about meaning. Iconic words, and especially onomatopoeias (Laing, 2019), may be learned earlier also because of the resemblance between word form and referent (Dingemanse et al., 2015; Nielsen & Dingemanse, 2020), offering a first source of information to figure out that words are used referentially in natural languages (Harnad, 1990). Our results suggest another possible advantage: Words with stronger form-meaning systematicity may occupy a privileged place in the language network, by which they have stronger relations with other words when considering both form and meaning. Crucially, it is not just a matter of occupying denser neighbourhoods, but of sharing similar relations across modalities. Our results call for more studies on the structure of the language network which consider cross-modal mappings, and its potential role in facilitating language learning.

Furthermore, we have shown that systematicity and iconicity are both reliable predictors of AoA and that they relate differently to SND; more iconic words tend to have lower SND, while the opposite was reported for systematicity, suggesting that they tap into different aspects of non-arbitrariness. At a fundamental level, however, FSC and iconicity both rely on analogies; iconicity draws attention on similarities between word form and perception, with words reproducing some physical property of their referent. Systematicity, on the contrary, draws attention on analogies between word form and lexical semantics. It may thus be the case that the risk of confusing two iconic words in context is higher when the two words are similarly iconic (which entails they have similar forms and mimic reality in a similar way), but lower when the two words are similarly systematic, since the situational context may help disambiguate them (hence the different relation with SND). These are however speculations at present and more work is needed to properly characterise the effects of iconicity and systematicity. Moreover, and more importantly, while iconicity can facilitate language learning from the very start of language acquisition, systematicity can only work after a few words are known. Therefore, we hypothesise that FSC and iconicity ultimately rely on a similar process whereby learners notice a reliable correspondence across two domains and use this to refine their intuitions about lexical meaning. First, this process would work on the observation that a word which resembles a referent in the world tends to denote that referent. Then, when some words are known, it could be extended to noticing that words that sound similar tend to refer to somewhat similar meanings. Future work should investigate the time course of the effect of both iconicity and systematicity on language learning: our study offers a promising way to conceptualise their impact on AoA and an effective tool to quantify systematicity, which has been validated in several studies on a variety of psycholinguistic phenomena.

Following existing work on non-arbitrariness (Monaghan et al., 2014; Perry et al., 2015), it could be further hypothesised that indeed later acquired words increase the degree of arbitrariness in the lexicon. Our work also suggests that systematicity computed over smaller vocabularies predicts AoA just as well or better than when computed on a larger vocabulary, confirming that larger vocabularies may feature less systematicity. Some studies also suggest that the role of systematicity is modulated by lexical knowledge (Brand et al., 2018; Monaghan et al., 2011). When few words are known, systematicity may actually offer cues to the specific semantic content of an individual word, whereas with larger vocabularies, systematicity may be more useful to derive a word’s category (lexical or semantic). Longitudinal computational analyses which leverage productive form-meaning mapping functions (Amenta et al., 2017; Baayen et al., 2019; Cassani et al., 2020; Chuang et al., 2021; Hendrix & Sun, 2020; Marelli et al., 2015) to map form onto meaning on the basis of the available lexicon can thus be useful to illuminate these dynamics further, by studying the semantic representations derived from word forms and how they relate with the available representations in semantic space at any given time. The present article shows that FSC measures hold promise in this regard since they have a strong unique relation with AoA, and they have been shown to work with words and pseudowords alike (Hendrix & Sun, 2020).

While our work focused on establishing whether FSC measures influenced AoA, our results only apply to English. This choice was primarily motivated by the large availability of rich secondary data on which to perform our computational analyses and the possibility of relying upon a large sample size. However, the effects we have investigated are not hypothesised to be language-specific (Blasi et al., 2016): Other languages have larger shares of iconic words in their vocabulary than English, e.g., Japanese. Therefore, replicating our study on a larger pool of typologically different languages will likely illuminate the relation between form-meaning systematicity and acquisition further.

Finally, our work did not provide conclusive evidence about the relation between systematicity, SND, and PND and how they may or may not interact in language learning. Interactions that proved significant in the main analysis did not prove reliable when using non-linear models or when predicting subjective AoA norms. More work should thus go into investigating these relations, formulating more precise and stringent hypotheses, which can build on the evidence we provide, particularly about the different nature of iconicity and systematicity, which we have previously discussed, and how this may bear relevance when analysing the relation between different forms of non-arbitrariness and other lexical properties.

In conclusion, our study showed that computational measures of form-meaning systematicity that rely on the coherence of local neighbourhoods in form and meaning account for a significant portion of variance in AoA. In detail, words with higher form-meaning coherence tend to be learned earlier. Our results thus show that English words for which it is easier to derive coherent semantic impressions from similar words tend to be learned earlier, suggesting a place in word learning for an analogical process which bridges word form and meaning, such that children could guesstimate the meaning of a novel word by relying on the meaning of known similar sounding words. Crucially, this effect is not reducible to words being highly entrenched in the language network when considering form and meaning alone. Moreover, the measures of form-meaning systematicity we used can be seamlessly extended to actually generate distributed semantic representations from form alone, allowing to study the role of form-meaning systematicity during development using large datasets and a data-driven approach.

## Supplemental Material

sj-pdf-1-qjp-10.1177_17470218211053472 – Supplemental material for Not just form, not just meaning: Words with consistent form-meaning mappings are learned earlier Click here for additional data file. Supplemental material, sj-pdf-1-qjp-10.1177_17470218211053472 for Not just form, not just meaning: Words with consistent form-meaning mappings are learned earlier by Giovanni Cassani and Niklas Limacher in Quarterly Journal of Experimental Psychology

## Notes

1. The number of character insertions, deletions, or substitutions needed to get from one word to another.

2. The distributional semantic space was trained on the concatenation of the UKWaC corpus (Ferraresi et al., 2008) and the subtlex corpus (Brysbaert & New, 2009), for a total of more than 2.3 billion tokens, using the Continuous Bag of Words (CBoW) algorithm from word2vec (CBoW, Mikolov et al., 2013; window size = 6, embedding dimensions = 300).

3. Explorations of different values (up to 50) for this parameter did not yield qualitatively different results, suggesting that reported patterns are robust to changes in the choice of *k*.

## References

[bibr1-17470218211053472] Abramova E. Fernández R. Sangati F. (2013). Automatic labeling of phonesthemic senses. In Proceedings of the 35th Annual Meeting of the Cognitive Science Society, Volume 35. Cognitive Science Society.

[bibr2-17470218211053472] Amenta S. Crepaldi D. Marelli M. (2020). Consistency measures individuate dissociating semantic modulations in priming paradigms: A new look on semantics in the processing of (complex) words. Quarterly Journal of Experimental Psychology, 73(10), 1546–1563. 10.1177/1747021820927663 32419617

[bibr3-17470218211053472] Amenta S. Hasenäcker J. Crepaldi D. Marelli M. (submitted). Prediction at the intersection of sentence context and word form: Evidence from eye-movements in reading. Psychonomic Bulletin & Review. 10.3758/s13423-022-02223-9 PMC10264485 36510092

[bibr4-17470218211053472] Amenta S. Marelli M. Sulpizio S. (2017). From sound to meaning: Phonology-to-semantics mapping in visual word recognition. Psychonomic Bulletin & Review, 24(3), 887–893. 27572870 10.3758/s13423-016-1152-0

[bibr5-17470218211053472] Baayen R. H. Chuang Y.-Y. Shafaei-Bajestan E. Blevins J. P. (2019). The discriminative lexicon: A uniﬁed computational model for the lexicon and lexical processing in comprehension and production grounded not in (de)composition but in linear discriminative learning. Complexity, 2019, Article 4895891.

[bibr6-17470218211053472] Baayen R. H. Piepenbrock R. Gulikers L. (1996). The CELEX Lexical Database (CD-ROM). Linguistic data consortium. University of Pennsylvania.

[bibr7-17470218211053472] Bates E. MacWhinney B. (1987). Competition, variation, and language learning. In MacWhinney B. (Ed.), Mechanisms of language acquisition: The 20th annual Carnegie Symposium on cognition book section 6 (pp. 157–193). Lawrence Erlbaum Associates.

[bibr8-17470218211053472] Bergen B. (2004). The psychological reality of phonaesthemes. Language, 80, 290–311.

[bibr9-17470218211053472] Blasi D. E. Wichmann S. Hammarstrom H. Stadler P. F. Christiansen M. H. (2016). Sound-meaning association biases evidenced across thousands of languages. Proceedings of the National Academy of Sciences, 113(39), 10818–10823. 10.1073/pnas.1605782113 PMC5047153 27621455

[bibr10-17470218211053472] Bojanowski P. Grave E. Joulin A. Mikolov T. (2017). Enriching word vectors with subword information. arXiv:1607.04606 [cs]. arXiv: 1607.04606.

[bibr11-17470218211053472] Braginsky M. Yurovsky D. Marchman V. A. Frank M. C. (2019). Consistency and variability in children’s word learning across languages. Open Mind (Camb), 3, 52–67. 31517175 10.1162/opmi_a_00026 PMC6716390

[bibr12-17470218211053472] Brand J. Monaghan P. Walker P. (2018). The changing role of sound-symbolism for small versus large vocabularies. Cognitive Science, 42(Suppl. 2), 578–590. 29235140 10.1111/cogs.12565 PMC6001752

[bibr13-17470218211053472] Brysbaert M. Biemiller A. (2017). Test-based age-of-acquisition norms for 44 thousand English word meanings. Behavior Research Methods, 49(4), 1520–1523. 27659480 10.3758/s13428-016-0811-4

[bibr14-17470218211053472] Brysbaert M. New B. (2009). Moving beyond Kučera and Francis: A critical evaluation of current word frequency norms and the introduction of a new and improved word frequency measure for American English. Behavior Research Methods, 41(4), 977–990. 19897807 10.3758/BRM.41.4.977

[bibr15-17470218211053472] Brysbaert M. Warriner A. B. Kuperman V. (2014). Concreteness ratings for 40 thousand generally known English word lemmas. Behavior Research Methods, 46(3), 904–911. 24142837 10.3758/s13428-013-0403-5

[bibr16-17470218211053472] Bullinaria J. A. Levy J. P. (2007). Extracting semantic representations from word co-occurrence statistics: A computational study. Behavior Research Methods, 39(3), 510–526. 17958162 10.3758/bf03193020

[bibr17-17470218211053472] Cassani G. Chuang Y.-Y. Baayen R. H. (2020). On the semantics of nonwords and their lexical category. Journal of Experimental Psychology. Learning, Memory, and Cognition, 46(4), 621–637. 31318232 10.1037/xlm0000747

[bibr18-17470218211053472] Christophe A. Guasti T. Nespor M. Dupoux E. Van Ooyen B. (1997). Reflections on phonological bootstrapping: Its role for lexical and syntactic acquisition. Language and Cognitive Processes, 12(5–6), 585–612.

[bibr19-17470218211053472] Chuang Y.-Y. Vollmer M. L. Shafaei-Bajestan E. Gahl S. Hendrix P. Baayen R. H. (2021). The processing of pseudoword form and meaning in production and comprehension: A computational modeling approach using linear discriminative learning. Behavior Research Methods, 53, 945–976. 32377973 10.3758/s13428-020-01356-w PMC8219637

[bibr20-17470218211053472] Clark E. V. (2017). Morphology in language acquisition. In Spencer A. Zwicky A. M. (Eds.), The handbook of morphology (pp. 374–389). John Wiley & Sons, Ltd.

[bibr21-17470218211053472] Coltheart M. Davelaar E. Jonasson J. T. Besner D. (1977). Access to the internal lexicon. In Dornic S. (Ed.), Attention and performance (pp. 535–555). Lawrence Erlbaum Associates.

[bibr22-17470218211053472] Dautriche I. Mahowald K. Gibson E. Christophe A. Piantadosi S. T. (2017). Words cluster phonetically beyond phonotactic regularities. Cognition, 163, 128–145. 28342382 10.1016/j.cognition.2017.02.001

[bibr23-17470218211053472] Delgado J. Pereira R. Ferreira M. F. Farinha-Fernandes A. Guerreiro J. C. Faustino B. Domingues M. Ventura P. (2020). Sound symbolism is modulated by linguistic experience. Revista da Associação Portuguesa de Linguística, 11(7), 137–150.

[bibr24-17470218211053472] de Saussure F. (1916). Course in general linguistics. McGraw-Hill.

[bibr25-17470218211053472] Dingemanse M. Blasi D. E. Lupyan G. Christiansen M. H. Monaghan P. (2015). Arbitrariness, iconicity, and systematicity in language. Trends in Cognitive Sciences, 19(10), 603–615. 26412098 10.1016/j.tics.2015.07.013

[bibr26-17470218211053472] Eco U. (1995). The search for the perfect language. Blackwell.

[bibr27-17470218211053472] Farmer T. Christiansen M. Monaghan P. (2006). Phonological typicality influences on-line sentence comprehension. Proceedings of the National Academy of Sciences of the United States of America, 103, 12203–12208. 16882728 10.1073/pnas.0602173103 PMC1567719

[bibr28-17470218211053472] Ferraresi A. Zanchetta E. Bernardini S. Baroni M. (2008). Introducing and evaluating ukWaC, a very large web-derived corpus of English. In Evert S. Kilgarriff A. Sharoff S. (Eds.), Proceedings of the 4th Web as Corpus Workshop (WAC-4), Marrakesh, Morocco.

[bibr29-17470218211053472] Firth J. R. (1957). Papers in linguistics, 1934–1951. Oxford University Press.

[bibr30-17470218211053472] Fitneva S. Christiansen M. Monaghan P. (2009). From sound to syntax: Phonological constraints on children’s lexical categorization of new words. Journal of Child Language, 36, 967–997. 19105858 10.1017/S0305000908009252

[bibr31-17470218211053472] Fourtassi A. Bian Y. Frank M. C. (2020). The growth of children’s semantic and phonological networks: Insight from 10 languages. Cognitive Science, 44(7), Article e12847. 10.1111/cogs.12847 32621305

[bibr32-17470218211053472] Gasser M. (2004). The origins of arbitrariness in language. In Proceedings of the 26th Annual Meeting of the Cognitive Science Society, Chicago, IL, United States.

[bibr33-17470218211053472] Ghyselinck M. Custers R. Brysbaert M. (2004). The effect of age of acquisition in visual word processing: Further evidence for the semantic hypothesis. Journal of Experimental Psychology. Learning, Memory, and Cognition, 30, 550–554. 14979824 10.1037/0278-7393.30.2.550

[bibr34-17470218211053472] Harnad S. (1990). The symbol grounding problem. Physica D: Nonlinear Phenomena, 42(1–3), 335–346.

[bibr35-17470218211053472] Harris Z. (1954). Distributional structure. Word, 10(2–3), 146–152.

[bibr36-17470218211053472] Hendrix P. Sun C. C. (2021). A word or two about nonwords: Frequency, semantic neighborhood density, and orthography-to-semantics consistency effects for nonwords in the lexical decision task. Journal of Experimental Psychology. Learning, Memory, and Cognition, 47, 157–183. 31999159 10.1037/xlm0000819

[bibr37-17470218211053472] Hills T. T. Maouene J. Riordan B. Smith L. B. (2010). The associative structure of language: Contextual diversity in early word learning. Journal of Memory and Language, 63(3), 259–273. 20835374 10.1016/j.jml.2010.06.002 PMC2936494

[bibr38-17470218211053472] Hills T. T. Maouene M. Maouene J. Sheya A. Smith L. B. (2009). Longitudinal analysis of early semantic networks: Preferential attachment or preferential acquisition? Psychological Science, 20(6), 729–739. 19470123 10.1111/j.1467-9280.2009.02365.x PMC4216730

[bibr39-17470218211053472] Hinton L. Nichols J. Ohala J. J. (1994). Sound symbolism. Cambridge University Press.

[bibr40-17470218211053472] Hockett C. F. (1960). The origin of speech. Scientiﬁc American, 203(3), 88–97. 14402211

[bibr41-17470218211053472] Imai M. Kita S. (2014). The sound symbolism bootstrapping hypothesis for language acquisition and language evolution. Philosophical Transactions of the Royal Society B: Biological Sciences, 369(1651), 20130298. 10.1098/rstb.2013.0298 PMC4123677 25092666

[bibr42-17470218211053472] Imai M. Kita S. Nagumo M. Okada H. (2008). Sound symbolism facilitates early verb learning. Cognition, 109(1), 54–65. 18835600 10.1016/j.cognition.2008.07.015

[bibr43-17470218211053472] Jones S. D. Brandt S. (2019). Do children really acquire dense neighbourhoods? Journal of Child Language, 46(6), 1260–1273. 31500682 10.1017/S0305000919000473

[bibr44-17470218211053472] Kantartzis K. Imai M. Evans D. Kita S. (2019). Sound symbolism facilitates long-term retention of the semantic representation of novel verbs in three-year-olds. Languages, 4(2), 21.

[bibr45-17470218211053472] Kantartzis K. Imai M. Kita S. (2011). Japanese sound-symbolism facilitates word learning in English-speaking children. Cognitive Science, 35, 575–586.

[bibr46-17470218211053472] Kelly M. H. (1992). Using sound to solve syntactic problems: The role of phonology in grammatical category assignments. Psychological Review, 99(2), 349–364. 1594729 10.1037/0033-295x.99.2.349

[bibr47-17470218211053472] Köhler W. (1947). Gestalt psychology (2nd ed.). Liveright Publishing Corporation.

[bibr48-17470218211053472] Kovic V. Plunkett K. Westermann G. (2010). The shape of words in the brain. Cognition, 114(1), 19–28. 19828141 10.1016/j.cognition.2009.08.016

[bibr49-17470218211053472] Kuperman V. Estes Z. Brysbaert M. Warriner A. (2014). Emotion and language: Valence and arousal affect word recognition. Journal of Experimental Psychology. General, 143, 1065–1081. 24490848 10.1037/a0035669 PMC4038659

[bibr50-17470218211053472] Kuperman V. Stadthagen-Gonzalez H. Brysbaert M. (2012). Age-of-acquisition ratings for 30,000 English words. Behavior Research Methods, 44(4), 978–990. 22581493 10.3758/s13428-012-0210-4

[bibr51-17470218211053472] Laing C. (2019). A role for onomatopoeia in early language: Evidence from phonological development. Language and Cognition, 11(2), 173–187.

[bibr52-17470218211053472] Levenshtein V. I. (1966). Binary codes capable of correcting deletions, insertions and reversals. Soviet Physics Doklady, 10, 707–710.

[bibr53-17470218211053472] Liu N. F. Levow G.-A. Smith N. A. (2018). Discovering phonesthemes with sparse regularization. In Proceedings of the Second Workshop on Subword/Character Level Models (pp. 49–54). Association for Computational Linguistics.

[bibr54-17470218211053472] Lockwood G. Hagoort P. Dingemanse M. (2016). How iconicity helps people learn new words: Neural correlates and individual differences in sound-symbolic bootstrapping. Collabra, 2(1), 7.

[bibr55-17470218211053472] Louwerse M. Qu Z. (2017). Estimating valence from the sound of a word: Computational, experimental, and cross-linguistic evidence. Psychonomic Bulletin & Review, 24(3), 849–855. 27562762 10.3758/s13423-016-1142-2 PMC5486854

[bibr56-17470218211053472] Mandera P. Keuleers E. Brysbaert M. (2017). Explaining human performance in psycholinguistic tasks with models of semantic similarity based on prediction and counting: A review and empirical validation. Journal of Memory and Language, 92, 57–78.

[bibr57-17470218211053472] Marelli M. Amenta S. (2018). A database of orthography-semantics consistency (OSC) estimates for 15,017 English words. Behavior Research Methods, 50(4), 1482–1495. 29372490 10.3758/s13428-018-1017-8

[bibr58-17470218211053472] Marelli M. Amenta S. Crepaldi D. (2015). Semantic transparency in free stems: The effect of orthography-semantics consistency on word recognition. Quarterly Journal of Experimental Psychology, 68(8), 1571–1583. 10.1080/17470218.2014.959709 25269473

[bibr59-17470218211053472] Marelli M. Baroni M. (2015). Affixation in semantic space: Modeling morpheme meanings with compositional distributional semantics. Psychological Review, 122(3), 485–515. 26120909 10.1037/a0039267

[bibr60-17470218211053472] Marelli M. Gagn’e C. L. Spalding T. L. (2017). Compounding as abstract operation in semantic space: Investigating relational effects through a large-scale, data-driven computational model. Cognition, 166, 207–224. 28582684 10.1016/j.cognition.2017.05.026

[bibr61-17470218211053472] Maurer D. Pathman T. Mondloch C. J. (2006). The shape of boubas: Sound–shape correspondences in toddlers and adults. Developmental Science, 9(3), 316–322. 16669803 10.1111/j.1467-7687.2006.00495.x

[bibr62-17470218211053472] Mikolov T. Sutskever I. Chen K. Corrado G. S. Dean J. (2013). Distributed representations of words and phrases and their compositionality. In Burges C. J. C. Bottou L. Welling M. Ghahramani Z. Weinberger K. Q. (Eds.), Advances in neural information processing systems 26 (pp. 3111–3119). Curran Associates, Inc.

[bibr63-17470218211053472] Monaghan P. Christiansen M. H. Chater N. (2007). The phonological-distributional coherence hypothesis: Cross-linguistic evidence in language acquisition. Cognitive Psychology, 55(4), 259–305. 17291481 10.1016/j.cogpsych.2006.12.001

[bibr64-17470218211053472] Monaghan P. Christiansen M. H. Fitneva S. A. (2011). The arbitrariness of the sign: Learning advantages from the structure of the vocabulary. Journal of Experimental Psychology. General, 140(3), 325–347. 21517205 10.1037/a0022924

[bibr65-17470218211053472] Monaghan P. Mattock K. Walker P. (2012). The role of sound symbolism in language learning. Journal of Experimental Psychology. Learning, Memory, and Cognition, 38, 1152–1164. 22468804 10.1037/a0027747

[bibr66-17470218211053472] Monaghan P. Shillcock R. C. Christiansen M. H. Kirby S. (2014). How arbitrary is language? Philosophical Transactions of the Royal Society B: Biological Sciences, 369(1651), 20130299. 10.1098/rstb.2013.0299 PMC4123678 25092667

[bibr67-17470218211053472] Morgan J. L. Demuth K. (2014). Signal to syntax: Bootstrapping from speech to grammar in early acquisition. Taylor & Francis.

[bibr68-17470218211053472] Motamedi Y. Murgiano M. Perniss P. Wonnacott E. Marshall C. Goldin-Meadow S. Vigliocco G. (2020). Linking language to sensory experience: Onomatopoeia in early language development. Developmental Science, 24, Article e13066. 10.1111/desc.13066 33231339

[bibr69-17470218211053472] Nielsen A. K. Dingemanse M. (2020). Iconicity in word learning and beyond: A critical review. Language and Speech, 64, 52–72. 32308121 10.1177/0023830920914339 PMC7961653

[bibr70-17470218211053472] Nygaard L. Cook A. Namy L. (2009). Sound to meaning correspondences facilitate word learning. Cognition, 112, 181–186. 19447384 10.1016/j.cognition.2009.04.001

[bibr71-17470218211053472] Otis K. Sagi E. (2008). Phonaesthemes: A corpus-based analysis. In Proceedings of the 30th Annual Meeting of the Cognitive Science Society, Volume 30. Cognitive Science Society.

[bibr72-17470218211053472] Perniss P. Vigliocco G. (2014). The bridge of iconicity: From a world of experience to the experience of language. Philosophical Transactions of the Royal Society B: Biological Sciences, 369(1651), 20130300. 10.1098/rstb.2013.0300 PMC4123679 25092668

[bibr73-17470218211053472] Perry L. K. Perlman M. Lupyan G. (2015). Iconicity in English and Spanish and its relation to lexical category and age of acquisition. PLOS ONE, 10(9), Article e0137147. 10.1371/journal.pone.0137147 PMC4560417 26340349

[bibr74-17470218211053472] Peters R. Borovsky A. (2019). Modeling early lexicosemantic network development: Perceptual features matter most. Journal of Experimental Psychology. General, 148(4), 763–782. 30973265 10.1037/xge0000596 PMC6461380

[bibr75-17470218211053472] Pimentel T. McCarthy A. D. Blasi D. Roark B. Cotterell R. (2019). Meaning to form: Measuring systematicity as information. In Proceedings of the 57th Annual Meeting of the Association for Computational Linguistics (pp. 1751–1764). Association for Computational Linguistics.

[bibr76-17470218211053472] Pinker S. (1984). Language learnability and language development. Harvard University Press.

[bibr77-17470218211053472] Ramachandran V. S. Hubbard E. M. (2001). Synaesthesia—A window into perception, thought and language. Journal of Consciousness Studies, 8(12), 3–34.

[bibr78-17470218211053472] R Core Team. (2020). R: A language and environment for statistical computing. R Foundation for Statistical Computing.

[bibr79-17470218211053472] Reilly J. Westbury C. Kean J. Peelle J. E. (2012). Arbitrary symbolism in natural language revisited: When word forms carry meaning. PLOS ONE, 7(8), Article e42286. 10.1371/journal.pone.0042286 PMC3412842 22879931

[bibr80-17470218211053472] Rescorla R. A. Wagner A. R. (1972). A theory of pavlovian conditioning: Variations in the effectiveness of reinforcement and nonreinforcement. In Black A. H. Prokasy W. F. (Eds.), Classical conditioning II: Current research and theory (pp. 497). Appleton-Century-Crofts.

[bibr81-17470218211053472] Roy B. C. Frank M. C. DeCamp P. Miller M. Roy D. K. (2015). Predicting the birth of a spoken word. Proceedings of the National Academy of Sciences, 112(41), 12663–12668. 10.1073/pnas.1419773112 PMC4611597 26392523

[bibr82-17470218211053472] Sánchez-Gutiérrez C. H. Mailhot H. Deacon S. H. Wilson M. A. (2018). MorphoLex: A derivational morphological database for 70,000 English words. Behavior Research Methods, 50(4), 1568–1580. 29124719 10.3758/s13428-017-0981-8

[bibr83-17470218211053472] Sapir E. (1929). A study in phonetic symbolism. Journal of Experimental Psychology, 12(3), 225–239.

[bibr84-17470218211053472] Sharpe V. Marantz A. (2017). Revisiting form typicality of nouns and verbs. The Mental Lexicon, 12(2), 159–180.

[bibr85-17470218211053472] Shillcock R. Kirby S. McDonald S. Brew C. (2001). Filled pauses and their status in the mental lexicon. In ISCA Tutorial and Research Workshop (ITRW) on Disﬂuency in Spontaneous Speech.

[bibr86-17470218211053472] Sidhu D. M. Pexman P. M. (2018a). Five mechanisms of sound symbolic association. Psychonomic Bulletin & Review, 25(5), 1619–1643. 28840520 10.3758/s13423-017-1361-1

[bibr87-17470218211053472] Sidhu D. M. Pexman P. M. (2018b). Lonely sensational icons: Semantic neighbourhood density, sensory experience and iconicity. Language, Cognition and Neuroscience, 33(1), 25–31.

[bibr88-17470218211053472] Sidhu D. M. Williamson J. Slavova V. Pexman P. M. (2021). An investigation of iconic language development in four datasets. Journal of Child Language. 10.1017/S0305000921000040 34176538

[bibr89-17470218211053472] Tamariz M. (2008). Exploring systematicity between phonological and context-cooccurrence representations of the mental lexicon. The Mental Lexicon, 3(2), 259–278.

[bibr90-17470218211053472] Tomaschek F. Hendrix P. Baayen R. H. (2018). Strategies for addressing collinearity in multivariate linguistic data. Journal of Phonetics, 71, 249–267.

[bibr91-17470218211053472] Tucker B. V. Brenner D. Danielson D. K. Kelley M. C. Nenadić F. Sims M. (2019). The massive auditory lexical decision (MALD) database. Behavior Research Methods, 51, 1187–1204. 29916041 10.3758/s13428-018-1056-1

[bibr92-17470218211053472] Turney P. D. Pantel P. (2010). From frequency to meaning: Vector space models of semantics. Journal of Artiﬁcial Intelligence Research, 37, 141–188.

[bibr93-17470218211053472] Venables W. N. Ripley B. D. (1999). Modern applied statistics with S-PLUS. Springer.

[bibr94-17470218211053472] Wikse Barrow C. Nilsson Bjorkenstam K. Strombergsson S. (2019). Subjective ratings of age-of-acquisition: Exploring issues of validity and rater reliability. Journal of Child Language, 46(2), 199–213. 30348232 10.1017/S0305000918000363

[bibr95-17470218211053472] Wood S. N. (2001). Mgcv: Gams and generalized ridge regression for r. R News, 1(2), 20–25.

[bibr96-17470218211053472] Wood S. N. (2017). Generalized additive models: An introduction with R. CRC Press.

[bibr97-17470218211053472] Wright M. N. Ziegler A. (2017). Ranger: A fast implementation of random forests for high dimensional data in C++ and R. Journal of Statistical Software, 77(1), 1–17.

[bibr98-17470218211053472] Wright Cassidy K. Kelly M. H. (2001). Children’s use of phonology to infer grammatical class in vocabulary learning. Psychonomic Bulletin & Review, 8(3), 519–523. 11700903 10.3758/bf03196187

[bibr99-17470218211053472] Yarkoni T. Balota D. Yap M. (2008). Moving beyond Coltheart’s N: A new measure of orthographic similarity. Psychonomic Bulletin & Review, 15(5), 971–979. 18926991 10.3758/PBR.15.5.971

