# Supplementary material for: Not just form, not just meaning: Words with consistent form-meaning mappings are learned earlier
Source: Q J Exp Psychol (Hove). 2021 Oct 21;75(8):1464–82. doi: 10.1177/17470218211053472 (PMC9245153; doi:10.1177/17470218211053472)
Supplement: sj-pdf-1-qjp-10.1177_17470218211053472 – Supplemental material for Not just form, not just meaning: Words with consistent form-meaning mappings are learned earlier [file sj-pdf-1-qjp-10.1177_17470218211053472.pdf]

Not just form, not just meaning: Words with consistent form-meaning mappings are learned earlier - Online Supplementary Material

Giovanni Cassani and Niklas Limacher

October 13, 2021

Appendix A: Non-linear models

Since our hypothesis only concerns a negative relation between form-meaning systematicity and AoA, such that more systematic words tend to be acquired earlier, we presented linear models in the main text. However, to ensure that any conclusion we draw does not depend on the linearity assumption, we also ran the analyses from the main text using Generalised Additive Models (GAMs, Wood, 2017), implemented using the *mgcv* package in R (Wood, 2001). We started by fitting a baseline statistical model including all control variables as smooths (keeping the default parameters). We then added a partial tensor product interaction between PND and SND and checked the Δ_{AIC} with respect to the simpler model. Since this term did not improve model fit ($\Delta_{AIC} = -0.502$), we used the simpler model as our baseline to compare models including PSC measures.

Table 1 provides the empirical degrees of freedom (edf), reference degrees of freedom (rdf), F statistic and associated p-value, as well as the Δ_{AIC} with respect to the baseline statistical model for PSC_{te} and PSC_{ld} (when added to the baseline statistical model). We can see that both measures reliably improve the model fit, suggesting that their predictive power is not tied to the assumption of linearity. We further observed that the effect of PSC_{ld} is roughly linear and negative, while that of PSC_{te} is rather u-shaped, with lower predicted AoA for words with average PSC scores. However, we also note that PSC_{te} leads to a much lower Δ_{AIC}, consistently with what we observed with the linear model, suggesting that the relaxation of the linearity assumption did not change the overall picture. We do not have at present a precise explanation for why the effect of PSC_{te} on AoA is u-shaped while that of PSC_{ld} is linear and negative: the former is unexpected given the hypothesis on the role of systematicity in language learning and indicates that retrieving neighbours based on whether or not they embed the target word may not appropriately capture the effect of systematicity on language acquisition. We expanded on this point in the discussion.

	edf	rdf	F	p	Δ_{AIC}
PSC_{te}	3.910	4.874	10.726	$< .001$	46.48
PSC_{ld}	4.312	5.380	55.080	$< .001$	285.02

Table 1: Unique effect of PSC objective AoA norms, using Generalised Additive Models (GAMs). The table displays empirical degrees of freedom (edf), reference degrees of freedom (rdf), F statistics, p values and difference in AIC with respect to the baseline statistical model including smooths for control variables (Δ_{AIC}).

PSC_{ld} further improved the model fit over the baseline model which include iconicity ($edf = 3.928$, $rdf = 4.916$, $F = 9.832$, $p < .001$, $\Delta_{AIC} = 38.45$), confirming a robust unique relation with AoA also after controlling for iconicity. The effect was largely linear, slightly evening out for higher values of PSC.

Finally, we replicated the analysis considering whether PSC_{ld} reliably interacts with SND in influencing acquisition patterns, by adding a partial tensor product involving PSC_{ld} and SND to the baseline statistical model. This partial tensor product did not improve model fit ($\Delta_{AIC} = -1.919$), unlike what was reported in the main analysis. It seems thus that when relaxing the linearity assumption, the non-linear terms for SND and PSC_{ld} account for all the variance that the linear interaction between these two variables accounted for.

Appendix B: PSC after randomly pairing word forms and semantic representations

In this appendix, we present the outcome of a second robustness check, that we carried out in order to ensure that any relation between PSC and AoA

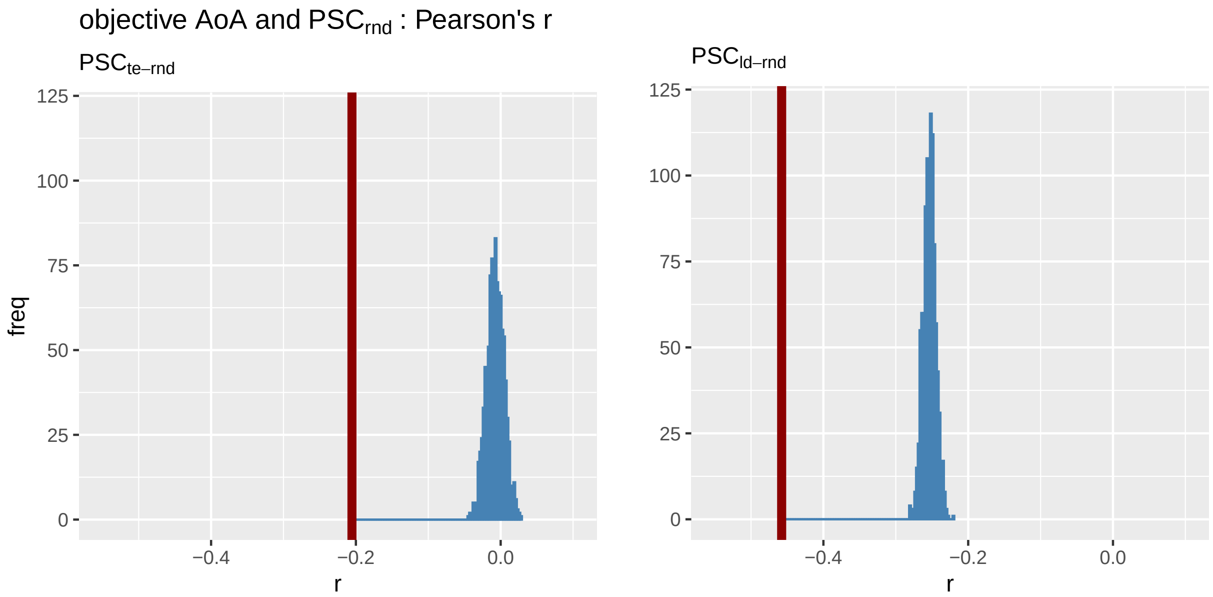

Figure 1: Distributions of Pearson's correlations between PSC measures computed on random shuffles of the semantic representations on the one hand, and objective AoA norms (top panels) or subjective AoA norms (bottom panels) on the other. The solid vertical line marks the correlation between AoA norms and PSC under the true form-meaning correspondences.

that we document in the article is not an epiphenomenon of other properties of the semantic space itself. Therefore, we implemented a random baseline where the correct pairings of word forms and lexical semantic representations are randomly scrambled, such that, e.g., the word form *dog* is no longer necessarily paired to the semantic representation of DOG but could be paired to any semantic representation. Crucially, in this analysis the form space and the semantic space remain unchanged, so the relations of proximity involving word representations are not altered, and PND and SND do not change. What changes is the relation between word form and lexical meaning, since the neighbours in phonological space now point to random words in semantic space. Therefore, this random baseline theoretically wipes out the form-meaning systematicity in the English lexicon: if an effect of PSC on AoA is still documented, it means that PSC is tapping into something different than expected. If its effect disappears, on the contrary, we would have further evidence that it indeed quantifies the degree to which form and meaning are consistent or not.

We randomly scrambled form-meaning correspondences 1000 times: for each iteration we computed the Pearson's correlation between PSC and AoA and the Δ_{AIC} between the baseline regression model, aoa_{base}, and both target regression models including PSC_{te} or PSC_{ld} computed from the random scrambling of form-meaning correspondences (to which we will refer as PSC_{te-rnd} and PSC_{ld-rnd} respectively). Importantly, SND and PND were unaffected and keep the same values they had in the main analysis. We thus derived a distribution of correlation values and Δ_{AIC} scores and computed p-values using a bootstrapping approach, as detailed in Equation 1.

$$p = \frac{1 + N(|t_{rnd}| >= |t_{obs}|)}{S + 1} \tag{1}$$

t_{rnd} indicates the statistic observed under a random shuffle of the semantic space, t_{obs} indicates the statistic observed under the true form-meaning correspondences, $|\cdot|$ indicates the absolute value, $N(\cdot)$ indicates the number of cases in which the logical condition is satisfied, and S indicates the number of random samples. Therefore, we compute how many statistics computed from random shuffles are more extreme than the observed one, smooth by adding 1 to avoid a p-value of 0, and normalise by the number of random samples (also smoothed by adding 1 to obtains a valid probability).

If PSC indeed captures something relevant about the acquisition process that is not simply an epiphenomenon of the structure of the underlying semantic space, we expect the distributions involving PSC_{te-rnd} and PSC_{ld-rnd} to be centered around 0, indicating that there is no reliable correlation nor

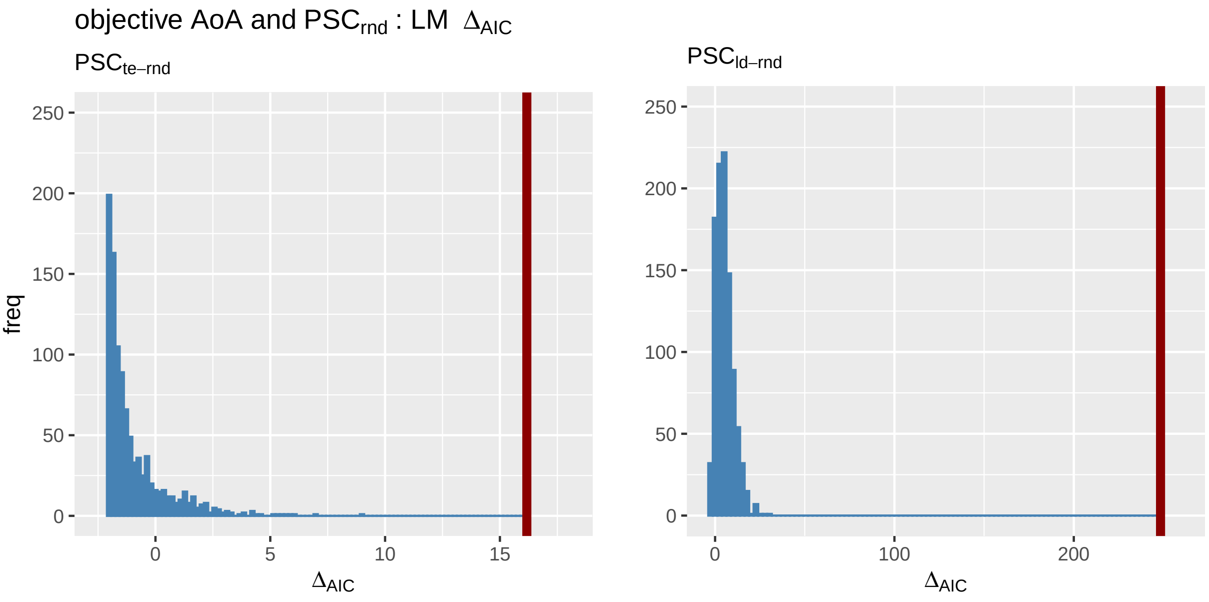

Figure 2: Distributions of Δ_{AIC} in linear regression models predicting objective (top panels) and subjective (bottom panels) AoA norms, including and excluding PSC measures computed on random shuffles of the semantic representations on the one hand, and objective AoA norms (top panels) or subjective AoA norms (bottom panels). The solid vertical line marks the Δ_{AIC} for the model using the true form-meaning correspondences.

any unique relation between PSC and AoA once true form-meaning correspondences are destroyed. If, on the contrary, we were to observe that the distributions involving PSC_{te-rnd} and PSC_{ld-rnd} are centered around the values observed for PSC_{te} and PSC_{ld}, we would conclude that the relation between PSC and AoA we observed is spurious. In Figure 1 we observe the distribution of correlation values between AoA on the one hand, and PSC_{te-rnd} and PSC_{ld-rnd} on the other, presenting the correlation observed for PSC_{te} and PSC_{ld} for reference. The same happens in Figure 2 for Δ_{AIC} scores computed for linear regression models.

In Figure 1, we see that the correlation involving PSC_{ld} and PSC_{te} is far more extreme (both $p <$ 0.001), in the predicted direction, than any correlation computed using PSC_{ld-rnd} and PSC_{te-rnd}. In some more detail, we observe a distribution centered around 0 for PSC_{te}, suggesting that this measure of form-meaning systematicity indeed does not capture any systematic pattern once form-meaning correspondences are destroyed. Quite surprisingly, however, PSC_{ld} shows a distribution centered around $r = -0.25$. If we go back to the pairwise correlation matrices, we observe that true PSC_{ld} measures have rather strong correlations with word length, frequency, and PND, suggesting that PSC_{ld} may pick up on other lexical properties.

However, when looking at the Δ_{AIC} distributions in Figure 2, the observed true statistic is reliably more extreme than statistics computed over random shuffles (again $p < .001$ for both PSC_{ld} and PSC_{te}). Moreover, the distributions for PSC_{ld-rnd} and PSC_{te-rnd} are close to 0, suggesting that once other variables are controlled for, no unique relation between PSC and AoA remains. This also hints to the fact that PSC captures something unique and different about AoA patterns than neighbourhood density measures in form and meaning do.

Appendix C: PSC from random subsets of the reference vocabulary

Since neighbourhood measures depend on the reference vocabulary, we ran a series of simulations to probe whether the relation between AoA and PSC is robust when the reference vocabulary used to retrieve nearest neighbours is smaller. Therefore, starting from the full reference vocabulary we used to derive the measures probed in the article, consisting of approximately 60K words from the SUBTLEX-US dataset, we created random subsets of the words, probing two different sampling rates. For each rate $r \in (50, 75)$, we carried out 500 it-

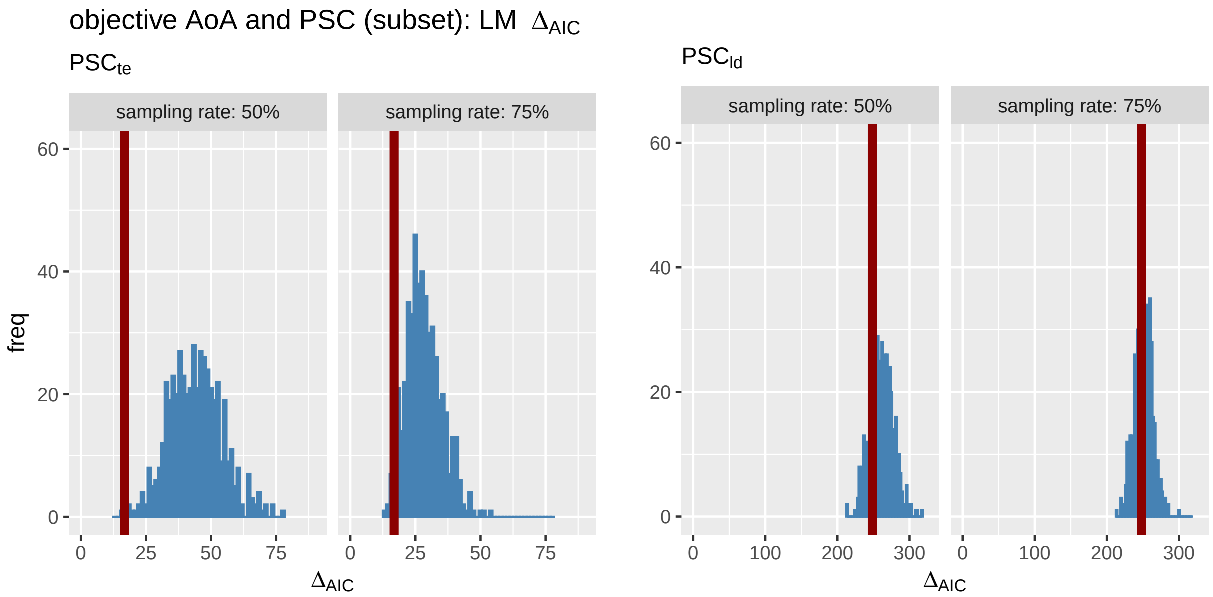

Figure 3: Distributions of Δ_{AIC} in linear regression models predicting objective (top panels) and subjective (bottom panels) AoA norms including and excluding PSC measures computed retrieving nearest neighbours in phonological space from random subsets (50% and 75%) of the reference lexicon. The solid vertical line marks the Δ_{AIC} for the model using the full reference vocabulary from SUBTLEX-US, consisting of approximately 60K words.

erations: in each iteration, we first sampled $r\%$ of the words randomly without replacement, then retrieved the nearest neighbours of the target words in phonological space in the vocabulary subset, proceeded to retrieve the corresponding semantic representations and computed PSC. In each iteration, we computed the Δ_{AIC} between the baseline regression model and the target regression models including PSC_{te} or PSC_{ld} computed from a random subset of the reference vocabulary. We thus derived a distribution of Δ_{AIC} values and computed p-values again using Eq 1.

In line with the procedure detailed in Appendix B for random baselines, if we observe that adding PSC_{te} and PSC_{ld} computed from random vocabulary subsets to the baseline statistical model does not improve model fit, we would conclude that the unique relation between PSC and AoA only holds for a large vocabulary. This would strongly challenge the usefulness of PSC as a useful construct to study the role of form-meaning systematicity in acquisition. On the contrary, if we observe that PSC_{te} and PSC_{ld} computed from vocabulary subsets improve model fit as much as, or even more than, their counterparts computed on the full vocabulary, we could conclude that the size and composition of the reference vocabulary used to retrieve nearest neighbours do not negatively affect the unique relation between AoA and PSC. In particular, if measures derived from smaller vocabularies would provide a better fit, we would have an encouraging, albeit tentative, indication that systematicity is stronger with smaller vocabularies, in line with the theory put forward by Gasser (2004) and with empirical evidence (Monaghan et al., 2014; Brand et al., 2018).

Figure 3 shows the distribution of Δ_{AIC} scores for PSC_{te} and PSC_{ld} computed on random sub-samples of the reference vocabulary. The vertical solid line indicates the Δ_{AIC} observed when considering the full reference vocabulary. The probability that the Δ_{AIC} for PSC_{te} computed on vocabulary subsets is larger than that computed on the full reference vocabulary is 0.998 when $r = 50\%$ and 0.972 when $r = 75\%$. For PSC_{ld}, the figures are 0.786 with $r = 50\%$ and 0.575 when $r = 75\%$. Therefore, the observed Δ_{AIC} of PSC_{ld} with the full vocabulary is indistinguishable from the Δ_{AIC} distribution computed from random subsets of the reference vocabulary, irrespective of the sampling rate. For PSC_{te}, the Δ_{AIC} observed under the full reference vocabulary is actually reliably worse (at $\alpha = 0.05$) than the Δ_{AIC} computed from random vocabulary subsets, again regardless of the sampling rate. This analysis confirms that the unique relation we documented

between PSC and AoA is not only found when leveraging a large reference vocabulary, but also appears when considering a smaller vocabulary.

Appendix D: Disentangling the effects of PSC, PND, and SND

As indicated by the correlation between AoA and PSC_{ld-rnd} as well as by the correlation matrix in Figure 5 of the main paper, PSC_{ld} correlates with other measures of neighbourhood density in phonological and semantic space. Even though the main analysis explicitly controls for PND and SND, finding a robust effect of PSC on AoA, and the analyses provided in Appendix B show how random baselines do not explain any additional variance in AoA after controlling for PND and SND (and other control variables), we ran an extra analysis to ensure that the effect of PSC on AoA is not an epiphenomenon of neighbourhood density measures.

First, we ran a Principal Component Analysis (PCA) including PND, SND, and PSC_{ld} to derive three orthogonal Principal Components (PCs) that capture the entire variance in the original measures while not being collinear. By inputting the PCs in a linear regression model which also includes control variables and checking the effect of each PC on AoA, we can see whether any PC influences AoA and how it relates to the original variables. If we can find a PC which, e.g., loads positively on PSC_{ld} and negatively on PND and SND and influences AoA such that words with higher scores on the PC (and thus also on PSC_{ld}) tend to be learned earlier, we can be more confident that the effect of PSC on AoA is not a simple by-product of neighbourhood density.

Table 2 shows the correlation matrix between the PCs and the original variables. PC1 loads positively on PSC_{ld} and PND, while negatively on SND reflecting the main dimension of variability which captures words found in dense phonological neighbourhoods and dense semantic neighbourhoods which display form-meaning systematicity. PC2 captures words found in dense semantic neighbourhoods but sparse phonological neighorhoods, with little influence from PSC_{ld}. Finally, PC3 disentangles PSC_{ld} and PND (with a weak relation to SND), capturing words with high systematicity found in sparse phonological neighbourhoods. This component allows us to test whether PSC captures something different than PND and crucially influences AoA: if this component reliably predicts AoA, and does so in the positive direction predicted based on its relation with PSC_{ld}, we would have evidence confirming the relation between PSC and AoA. Table 3 shows the regression coefficients for the three PCs in predicting AoA while including the other control variables, providing the b coefficient, the corresponding standard error se, t statistic and p-value.

	Objective AoA		
	PC1	PC2	PC3
PSC_{ld}	0.90	-0.10	-0.41
PND	0.83	-0.42	0.37
SND	-0.51	-0.85	-0.14

Table 2: Correlation coefficients between principal components extracted from neighbourhood measures and PSC_{ld} and the original variables. PC1, PC2, and PC3 indicate the extracted principal components.

Measure	b	se	t	p
PC1	-0.536	0.043	-12.376	$< .001$
PC2	0.074	0.038	1.913	0.0558
PC3	0.515	0.061	8.440	$< .001$

Table 3: Regression coefficients for the principal components extracted from neighbourhood measures and PSC_{ld} on objective AoA norms. The table provides the b regression coefficient, the corresponding standard error (se), t statistic and p values.

We see that PC2 does not reliably predict AoA, while both PC1 and PC3 do. Interestingly, PC2 is the component with no relation to PSC_{ld}. PC1, unsurprinsigly, has a negative relation with AoA, showing that words found in dense phonological and semantic neighbourhood that also exhibit higher form-meaning systematicity tend to be learned earlier. More interestingly, PC3 also has a reliable and positive relation with AoA, despite its positive relation with PND and in line with its negative relation with PSC_{ld}. This component therefore highlights how PSC reliably predicts AoA in the expected direction, with less systematic words being learned later, regardless of the influence that PND has on AoA.

Appendix E: Orthography-to-Semantics Consistency

In order to ensure that patterns reported for form-meaning systematicity do not depend on the phonological encoding, we replicated the analyses in the ar-

Measure	β	se	t	p	Δ_{AIC}
OSC_{te}	-0.147	0.033	-4.478	$< .001$	18.05
OSC_{ld}	-0.529	0.046	-11.552	$< .001$	130.49

Table 4: Unique effect of OSC on objective AoA norms. The table displays regression coefficients (β) with associated standard errors (se), t statistics, p values and difference in AIC with respect to the corresponding baseline statistical model (Δ_{AIC}).

ticle considering orthographic forms. A few changes to the pipeline were made to ensure consistency. First, the length in phonemes was replaced with the length in letters. In a similar vein, we replaced PND with OLD20 (Yarkoni et al., 2008), used as a measure of orthographic neighbourhood density and computed using the OLD20 Python package[1]. We also did not filter the set of target words to avoid words for which no phonological representation was available in CELEX, which resulted in a larger sample of 7,930 words for the general analysis and 1,999 words for the analysis which controlled for iconicity. The orthographic counterpart to PSC, Orthography-to-Semantic Consistency (OSC), was computed following the same approach, considering an implementation where nearest orthographic neighbours are words whose orthographic form embeds that of the target word (OSC_{te}) and one in which nearest orthographic neighbours are words with the lowest Levenshtein distance to the target when considering orthographic word forms (OSC_{ld}). The reference vocabulary to retrieve neighbours remained the same.

We first report the correlation matrix (Figure 9), then test whether the interaction between SND and OLD20 is reliable. After determining the best fitting and most parsimonious model, we include OSC_{te} and OSC_{ld} and check whether they improve model fit by checking the Δ_{AIC} with respect to the baseline statistical model. We do the same with the subset of words for which iconicity ratings are available.

AoA has sizable correlations with all variables, including negative correlations with OSC measures: words with higher OSC tend to be acquired earlier, in line with what we observed for PSC. The correlation between OSC_{te} and AoA appears to be considerably smaller ($r(OSC_{te}) = -0.19$) than the correlation between PSC_{ld} and AoA ($r(OSC_{ld}) = -0.39$). Measures based on phonological transcriptions appeared to have a slightly stronger relation with AoA, although the difference is small, confirming that the

[1]Available at `https://github.com/stephantul/old20`.

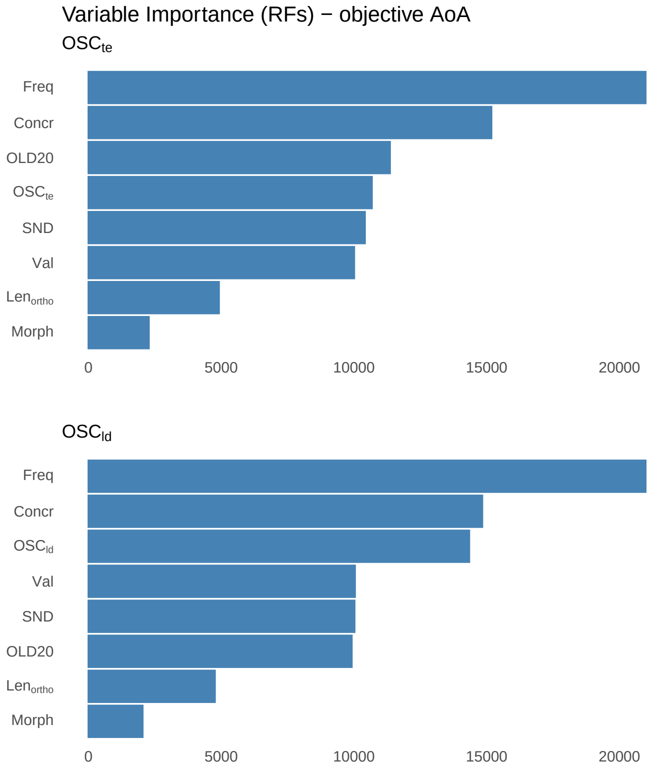

Figure 4: Variable importance plots from RF regressions for models including OSC measures on top of the baseline statistical model, predicting objective AoA norms. *Freq*: frequency; *Concr*: concreteness; *Val*: valence; *SND*: Semantic Neighbourhood Distance; Len_{ortho}: length in letters; *OLD20*: Orthographic Levenshtein Distance (k=20); *Morph*: morphological complexity (binary).

relation between AoA and FSC is robust to changes in the encoding of word form.

We also observed the predicted relations involving AoA and OLD20, with early acquired words being found in denser orthographic neighbourhoods. It is worth stressing that OLD20 is a measure of distance between target and neighbours, hence it is higher when the orthographic neighbours are further from the target. The same risk of collinearity detected for length in phonemes and PND appears here for word length in letters and OLD20, warranting the replication of the analysis using RF regression.

The interaction term involving OLD20 and SND turned out not to improve model fit ($\Delta_{AIC} = -1.069$). Table 4 provides the regression coefficients for OSC_{te} and OSC_{ld} when added to the baseline statistical model, which thus only includes the linear combination of the control variables.

OSC measures confirm the unique relation between form-meaning systematicity and acquisition

that has been documented in the article. OSC_{ld}, much like PSC_{ld}, improves model fit more and has a larger β coefficient than OSC_{te}, suggesting a stronger effect. Figure 4 shows the variable importance under RF regression: results are remarkably close to those observed for PSC, with OSC_{ld} ranking just below concreteness in terms of importance in predicting subjective AoA.

We then controlled for the confounding effect that iconicity may have on the relation between systematicity and AoA. OSC_{ld} still reliably improved model fit (Δ_{AIC} = 16.40), and its regression coefficient is reliably different from 0 in the predicted direction, with more systematic words being learned earlier even once the effect of iconicity is controlled for ($\beta = -0.304$, $se = 0.071$, $t = -4.289$, $p < .001$). Figure 5 further confirms that OSC tends to have high feature importance in RF regression also when controlling for iconicity.

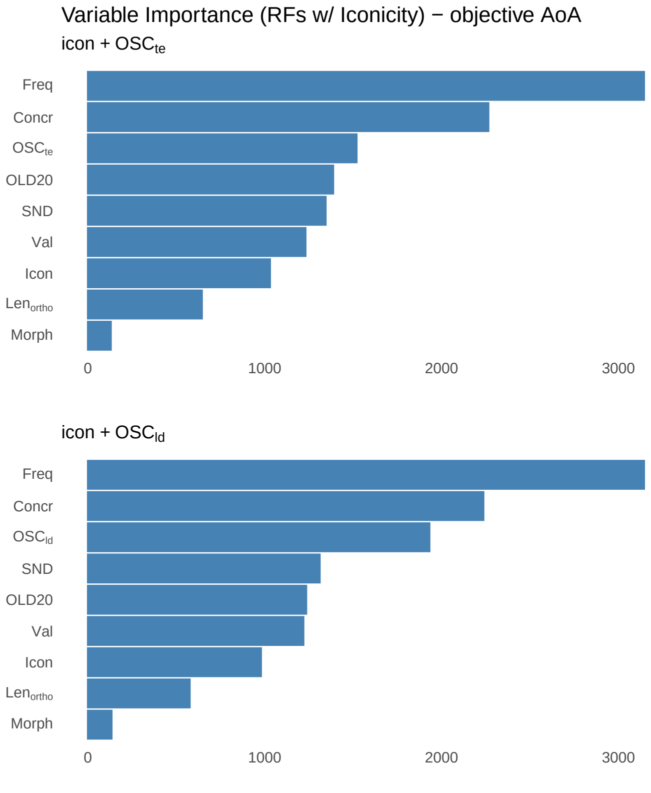

Figure 5: Variable importance plots from RF regressions for models including OSC measures on top of the baseline statistical model including iconicity, predicting objective AoA norms. *Freq*: frequency; *Concr*: concreteness; *Val*: valence; *SND*: Semantic Neighbourhood Distance; Len_{ortho}: length in letters; *OLD20*: Orthographic Levenshtein Distance (k=20); *Morph*: morphological complexity (binary); *Icon*: iconicity.

To conclude, we also found a reliable negative correlation between OSC_{ld} and SND ($r = -0.29[-0.31; -0.27]$, $t = -26.83$, $df = 7,928$, $p < .001$). We also fitted a linear model to predict OSC_{ld} as a linear combination of length in letters, OLD20, frequency, and AoA (Sidhu, Pexman, 2018). The β coefficient of SND was reliably different from 0 and in the predicted direction ($\beta = -0.23$, $se = 0.008$, $t = -28.64$, $p < .001$), in line with what was observed with PSC_{ld}. Again in line with results obtained when investigating PSC_{ld}, including an interaction between OSC_{ld} and SND did improve model fit when predicting AoA ($\Delta_{AIC} = 11.554$), and the regression coefficient of the target interaction was significant ($\beta = -0.103$, $se = 0.028$, $t = -3.681$, $p = .001$). However, the analysis reported in Appendix A invites caution when interpreting this interaction, whose qualitative pattern is in line with the one reported in Figure 5 of the main paper, with OSC_{ld} having a stronger negative effect on AoA for words found in sparser semantic neighbourhoods.

The code to replicate the robustness checks in Appendices A, B, C, and D also for OSC is available at the GitHub repository linked to this work: all analyses confirm the general pattern of a reliable unique relation between OSC and AoA, which is not affected by relaxing the linearity assumption, is only found when considering true form-meaning correspondences, is robust to changes in the reference vocabulary, and cannot be ascribed to an epiphenomenon of neighbourhood measures.

Appendix F: Predicting Subjective AoA norms

We also considered if systematicity predicts subjective AoA norms, taken from the dataset provided by Kuperman et al. (2012), who asked participants in a crowd-sourcing experiment when they thought they were first able to understand a word. Recent studies have highlighted how subjective judgments are confounded by other variables (Wikse Barrow et al., 2019). Therefore, it is interesting to check whether salient differences emerge with respect to the analysis of objective AoA norms or whether systematicity shows a similar relation with acquisition patterns probed objectively or subjectively. Moreover, subjective AoA norms have a more continuous distribution, which can help us probe whether reported effects are due to the peculiar, step-wise nature of objective AoA norms. After establishing

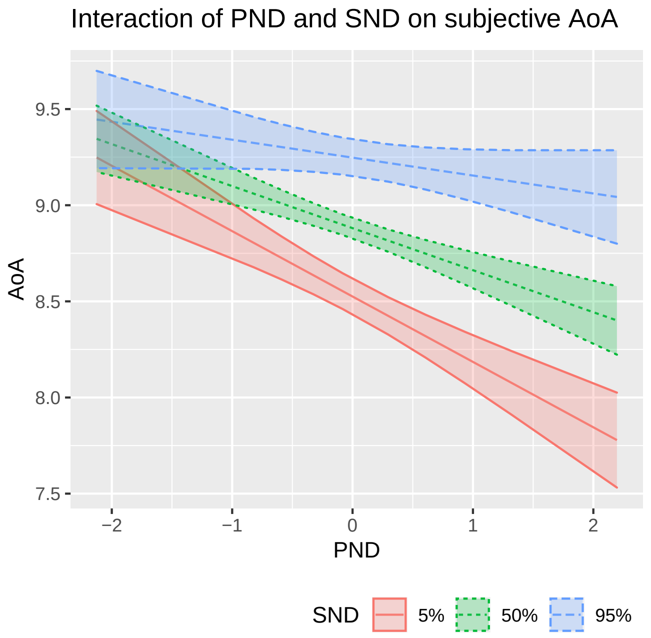

Figure 6: Interaction effects of Phonological Neighbourhood Density (PND) with Semantic Neighbourhood Distance (SND) on subjective AoA norms.

in Appendix E that OSC exhibits similar patterns to PSC, we show this analysis only for PSC and we replicate the statistical analyses we describe in the paper. Code to carry out this analysis for OSC is available on the GitHub repository of the project.

The correlation matrix in Figure 10 shows a very similar picture as that in Figure 2 in the main paper, with sizable correlations between PSC and subjective AoA norms. We see that PSC_{ld} has a stronger relation with AoA than PSC_{te}. We then checked whether the interaction between SND and PND improved the model fit to establish the baseline statistical model. When predicting subjective AoA, this interaction was significant, with a stronger negative effect of PND on AoA for words found in denser semantic neighbourhoods, as shown in Figure 6. We

Measure	β	se	t	p	Δ_{AIC}
PSC_{te}	-0.061	0.023	-2.619	$< .01$	4.87
PSC_{ld}	-0.625	0.031	-20.400	$< .001$	402.57

Table 5: Unique effect of PSC on AoA for subjective AoA norms. The table displays regression coefficients (β) with associated standard errors (se), t statistics, p values and difference in AIC with respect to the corresponding baseline statistical model (Δ_{AIC}).

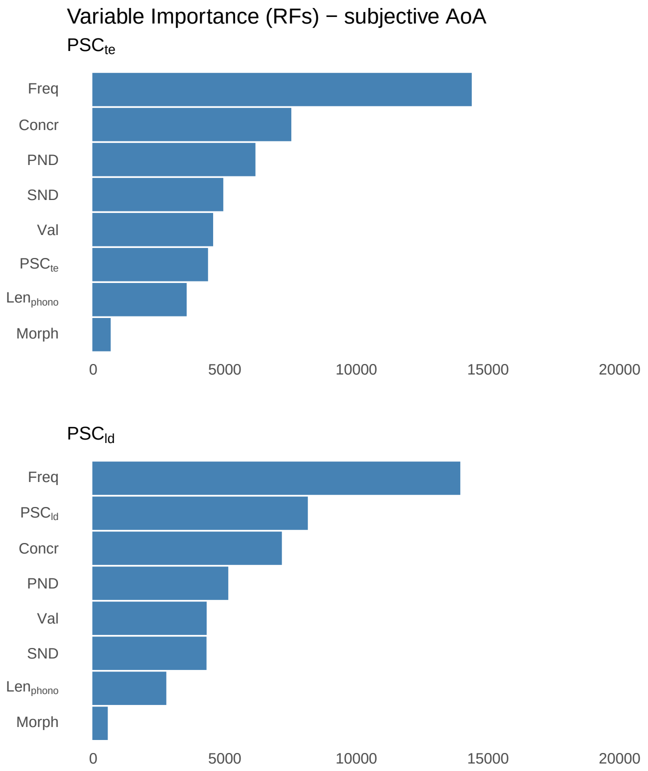

Figure 7: Variable importance plots from RF regressions for models including PSC measures on top of the baseline statistical model, predicting subjective AoA norms. *Freq*: frequency; *Concr*: concreteness; *Val*: valence; *SND*: Semantic Neighbourhood Distance; Len_{phono}: length in phonemes; *PND*: Phonological Neighbourhood Density; *Morph*: morphological complexity (binary).

therefore used the model including the interaction term as the baseline to which PSC measures were added.

Table 5 provides the regression coefficients for PSC_{ld} and PSC_{te} when predicting subjective AoA norms. We see the usual pattern emerging, with both variables improving the model fit, but with the predictive power of PSC_{ld} trumping that of PSC_{te}: both variables have a negative effect on AoA, but the β coefficient for the former is 10 times stronger than the latter.

The picture is consistent with the main analysis also when running RF regression: the r^2 improves by 0.032 points for PSC_{te} and by 0.061 for PSC_{ld} over the corresponding baseline RF regression. Variable importance is also consistent, with PSC_{ld} being among the most useful variables when predicting subjective AoA (see Figure 7).

PSC_{ld} reliably predicts subjective AoA ($\beta = -0.430$, $se = 0.055$, $t = -7.858$, $< .001$, $\Delta_{AIC} =$

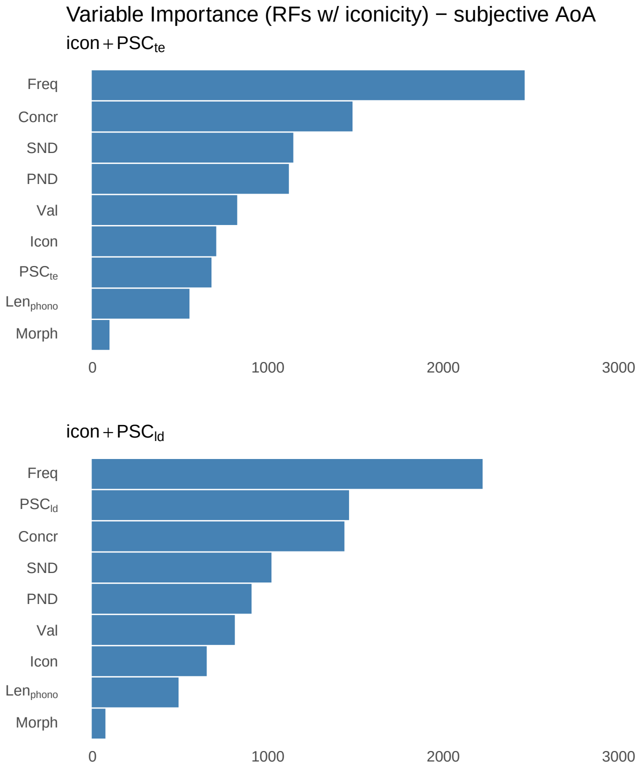

Figure 8: Variable importance plots from RF regressions for models including PSC measures on top of the baseline statistical model including iconicity, predicting subjective AoA norms. *Freq*: frequency; *Concr*: concreteness; *Val*: valence; *SND*: Semantic Neighbourhood Distance; Len_{phono}: length in phonemes; *PND*: Phonological Neighbourhood Density; *Morph*: morphological complexity (binary); *Icon*: iconicity.

59.08) even after controlling for iconicity, and is found among the most important variables as evidenced by the variable importance in RF regression (see Figure 8).

Finally, we checked whether an interaction between SND and PSC_{ld} reliably predicted AoA as reported for objective AoA norms. However, this interaction did not improve the model fit and the interaction term did not reach significance, confirming that this interaction, documented for objective AoA in the article but not when using GAMs (Appendix A) and here, may not be robust and should be interpreted with caution given current evidence.

References

Brand James, Monaghan Padraic, Walker Peter. The Changing Role of Sound-Symbolism for Small Versus Large Vocabularies // Cognitive Science. 2018. 42 Suppl 2. 578–590.

Gasser Michael. The Origins of Arbitrariness in Language // Proceedings of the 26th Annual Meeting of the Cognitive Science Society. 2004. 26, 26.

Kuperman Victor, Stadthagen-Gonzalez Hans, Brysbaert Marc. Age-of-acquisition ratings for 30,000 English words // Behavior Research Methods. XII 2012. 44, 4. 978–990.

Monaghan Padraic, Shillcock Richard C., Christiansen Morten H., Kirby Simon. How arbitrary is language? // Philosophical Transactions of the Royal Society B: Biological Sciences. IX 2014. 369, 1651. 20130299.

Sidhu David M., Pexman Penny M. Lonely sensational icons: semantic neighbourhood density, sensory experience and iconicity // Language, Cognition and Neuroscience. I 2018. 33, 1. 25–31.

Wikse Barrow Carla, Nilsson Bjorkenstam Kristina, Strombergsson Sofia. Subjective ratings of age-of-acquisition: exploring issues of validity and rater reliability // Journal of Child Language. 2019. 46, 2. 199–213.

Wood Simon N. mgcv: GAMs and generalized ridge regression for R // R news. 2001. 1, 2. 20–25.

Wood Simon N. Generalized additive models: an introduction with R. 2017.

Yarkoni Tal, Balota David, Yap Melvin. Moving beyond Coltheart's N: A new measure of orthographic similarity // Psychonomic Bulletin & Review. X 2008. 15, 5. 971–979.

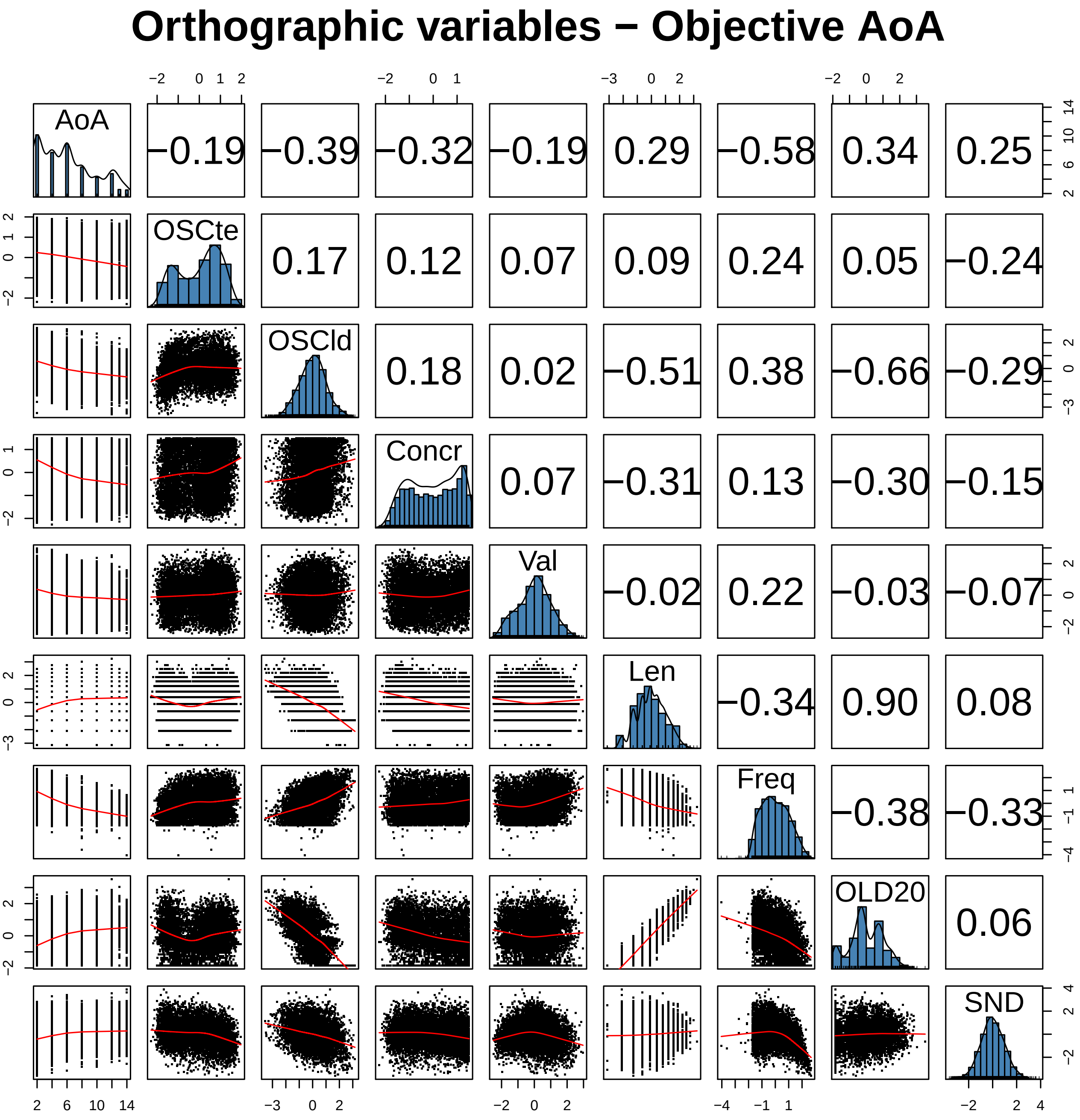

Figure 9: Pairwise Pearson's correlations involving independent variables and AoA for orthographic variables. The main diagonal provides histograms showing the distribution of each variable; the upper triangle provides correlation coefficients, the lower triangle provides scatterplots, where the red line is a LOESS fit. *Freq*: frequency; *Concr*: concreteness; *Val*: valence; *SND*: Semantic Neighbourhood Distance; *Len*: length in letters; *OLD20*: Orthographic Levenshtein Distance (k = 20).

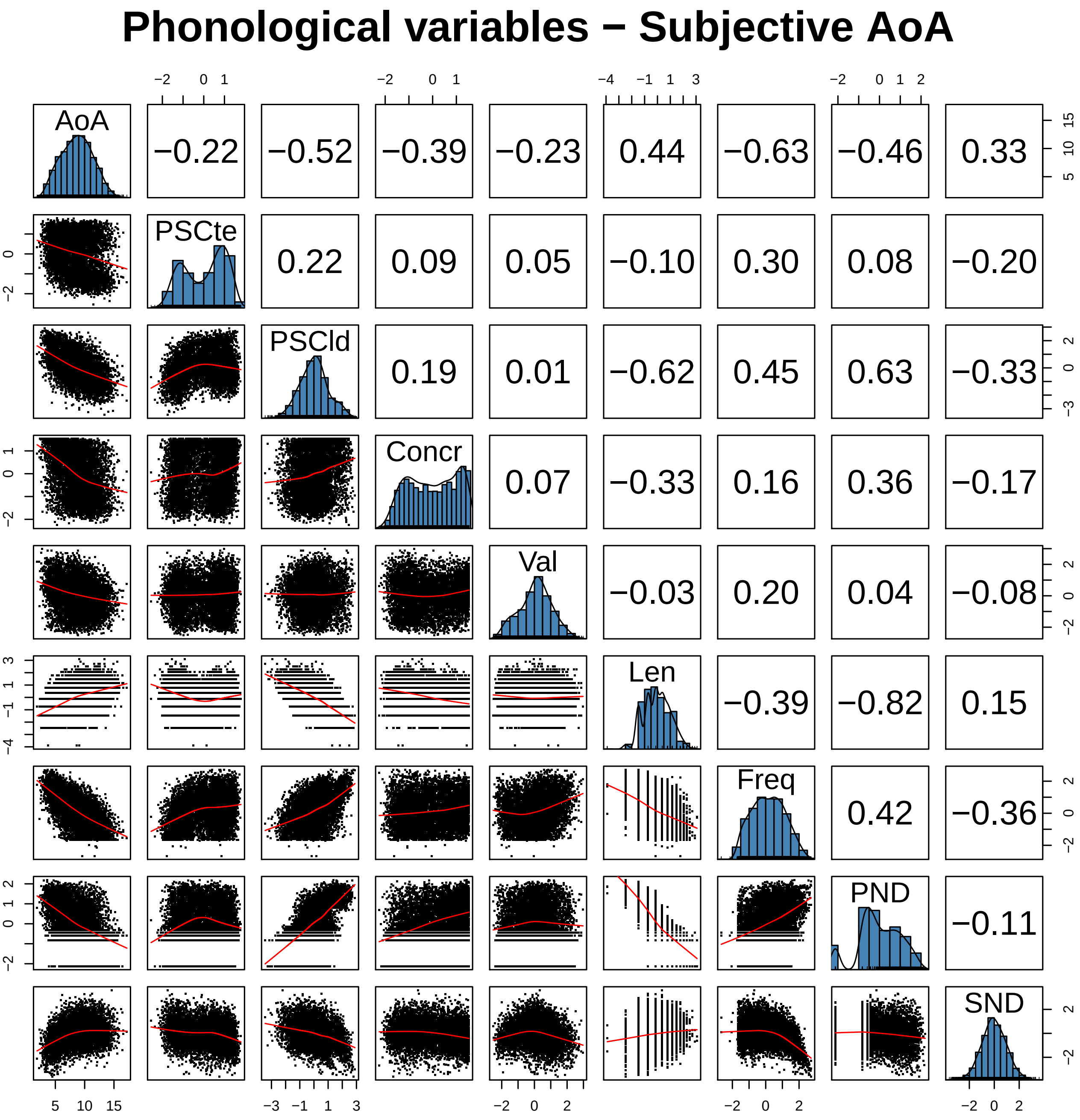

Figure 10: Pairwise Pearson's correlations involving independent variables and subjective AoA norms for phonological variables. The main diagonal provides histograms showing the distribution of each variable; the upper triangle provides correlation coefficients, the lower triangle provides scatterplots, where the red line is a LOESS fit. *Freq*: frequency; *Concr*: concreteness; *Val*: valence; *SND*: Semantic Neighbourhood Distance; *Len*: length in phonemes; *PND*: Phonological Neighbourhood Density.
